# Apical-Basal Polarity Signaling Components, Lgl1 and aPKCs, Control Glutamatergic Synapse Number and Function

**DOI:** 10.1016/j.isci.2019.09.005

**Published:** 2019-09-09

**Authors:** John Scott, Sonal Thakar, Ye Mao, Huaping Qin, Helen Hejran, Su-Yee Lee, Ting Yu, Olga Klezovitch, Hongqiang Cheng, Yongxin Mu, Sourav Ghosh, Valeri Vasioukhin, Yimin Zou

**Affiliations:** 1 Neurobiology Section, Biological Sciences Division, University of California, San Diego, La Jolla, CA 92093, USA; 2 Division of Human Biology, Fred Hutchinson Cancer Research Center, Seattle, WA 98109, USA; 3 Department of Neurology, Yale University, New Haven, CT 06511, USA; 4 Department of Medicine, University of California, San Diego, La Jolla, CA 92093, USA

**Keywords:** Biological Sciences, Cell Biology, Cellular Neuroscience, Neuroscience

## Abstract

Normal synapse formation is fundamental to brain function. We show here that an apical-basal polarity (A-BP) protein, Lgl1, is present in the postsynaptic density and negatively regulates glutamatergic synapse numbers by antagonizing the atypical protein kinase Cs (aPKCs). A planar cell polarity protein, Vangl2, which inhibits synapse formation, was decreased in synaptosome fractions of cultured cortical neurons from *Lgl1* knockout embryos. Conditional knockout of *Lgl1* in pyramidal neurons led to reduction of AMPA/NMDA ratio and impaired plasticity. *Lgl1* is frequently deleted in Smith-Magenis syndrome (SMS). Lgl1 conditional knockout led to increased locomotion, impaired novel object recognition and social interaction. *Lgl1+/−* animals also showed increased synapse numbers, defects in open field and social interaction, as well as stereotyped repetitive behavior. Social interaction in *Lgl1+/−* could be rescued by NMDA antagonists. Our findings reveal a role of apical-basal polarity proteins in glutamatergic synapse development and function and also suggest a potential treatment for SMS patients with *Lgl1* deletion.

## Highlights

• Lgl1 regulates glutamatergic synapse numbers by inhibiting aPKC
• *Lgl1 cKO* leads to reduced AMPA/NMDA ratio in development and adulthood
• *Lgl1* conditional knockout impairs novel object cognition and social interaction
• Social interaction deficits can be rescued by NMDA receptor blockade

## Graphical Abstract

**Figure:**
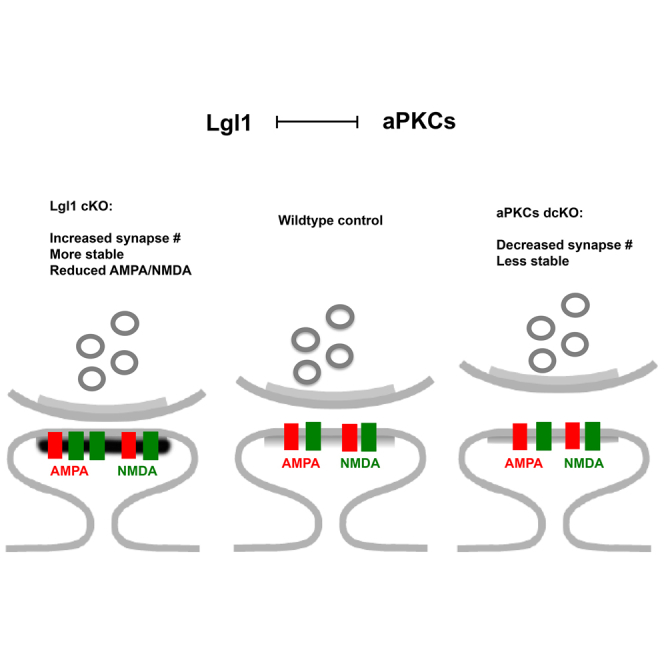

## Introduction

Glutamatergic synapses are the major class of excitatory synapses in the mammalian central nervous system (Collingridge et al., 1983, Monaghan et al., 1989, Watkins and Evans, 1981). Normal development and plasticity of glutamatergic synapses are essential to the emergence of normal behavioral functions, the disruption of which causes various neurological and neuropsychiatric disorders. A recent study showed that components of planar cell polarity (PCP) signaling pathway are key regulators of glutamatergic synapse formation (Thakar et al., 2017). Celsr3 is essential for glutamatergic synapse formation, whereas Vangl2 negatively regulates glutamatergic synapse formation. Therefore, PCP signaling components can both positively and negatively regulate glutamatergic synapse numbers.

Lethal giant larvae (Lgl1) is a key component of the highly conserved apical-basal polarity signaling pathway, which polarizes epithelial cells along the apical and basolateral axis (Karner et al., 2006). Lgl1 forms the basolateral complexes with Scribble and Discs Large (Dlg) and mutually excludes and antagonizes the function of the apical complex, the aPKC/Par3/Par6 complex. Lgl1 has been implicated in polarized exocytosis and is essential for establishing or maintaining apical-basal polarity (Betschinger et al., 2003, Georgiou et al., 2008, Macara, 2004, Yamanaka et al., 2003, Yamanaka et al., 2006). Dlg homologs are important postsynaptic scaffold proteins, called *MAGUKs* (Zhu et al., 2016). MAGUK proteins play essential roles in postsynaptic density organization and glutamate receptor trafficking and clustering. In addition, Lgl1 has been shown to associate and co-traffic with FMRP (Zarnescu et al., 2005), a translational regulator of many synaptic components. Apical-basal and planar polarity pathways are known to interact with each other. For example, apical-basal polarity signaling has been recently shown to regulate the location of PCP signaling (Chuykin et al., 2018).

Here we report that conditional knockout of *Lgl1* from postnatal day 7 (P7) in hippocampal pyramidal neurons, before the peak of glutamatergic synapse formation, resulted in increased glutamatergic synapse density. In *aPKC* double conditional knockout (KO) (dcKO), glutamatergic synapse numbers were initially normal (at P14) but were reduced in adulthood. *Triple conditional KO (tcKO)* of *Lgl1* and *aPKCs* rescued the defects of synapse numbers in *Lgl1 cKO*. In the synaptosome fraction of cultured *Lgl1−/−* neurons Vangl2 was found decreased. *Lgl1 cKO* and *aPKC dcKO* led to opposite changes in ultrastructure, with loss of Lgl1 leading to a larger postsynaptic density and smaller synaptic cleft. *Lgl1 cKO* also showed reduced AMPA/NMDA receptor ratio. Deleting Lgl1 in adulthood also led to increased synapse numbers and a much greater reduction of AMPA/NMDA receptor ratio, as well as deficit in long-term potentiation. These results suggest that Lgl1 regulates glutamate receptor trafficking, potentially through its binding partners, the MAGUK proteins. Therefore, together with PCP signaling, apical-basal signaling also has a profound influence on synapse formation, forming another layer of regulation, potentially allowing additional regulatory inputs.

*Lgl1* is frequently deleted in a chromosome 17 p11.2 microdeletion disorder, called Smith-Magenis syndrome (SMS) (Smith et al., 1986). SMS is a *de novo* genetic disorder arising very early in embryonic development through homologous recombination (Chen et al., 1997). A deletion interval of 3.5 Mb occurs in approximately 70% of patients (Gropman et al., 2007). Individuals with the deletion are frequently diagnosed with autism spectrum disorders (ASDs), attention-deficit/hyperactivity disorder, obsessive-compulsive disorder (OCD), or other behavioral disorders (Dykens et al., 1997, Dykens and Smith, 1998, Laje et al., 2010, Martin et al., 2006, Smith et al., 1998). Symptoms vary between individuals despite common deletions (Edelman et al., 2007, Potocki et al., 2003), and multiple genes likely contribute to the syndrome (Girirajan et al., 2006). Recent work has implicated *Rai1* in non-ASD symptoms of SMS (Huang et al., 2016). The locus for *Lgl1* lies within a refined consensus deletion site of ∼950 kb for SMS that has been reported in genetic studies of patients carrying the chromosomal deletion (Vlangos et al., 2003). Fluorescence *in situ* hybridization probes targeting the sequence for the human homolog of *Lgl1* fail to hybridize with one of the copies of chromosome 17 in patients with SMS (Koyama et al., 1996). The functional role of *Lgl1* in SMS has not been reported.

We found that *Lgl1* conditional KO in excitatory neurons from early postnatal stages (P7) resulted in behavioral deficits in adulthood, such as hyperactivity, cognitive impairment, and social interaction defects. We found that *Lgl1+/−* animals also had higher synapse numbers and showed impaired social interaction and increased stereotyped repetitive behaviors. Patients with SMS show either seizures or abnormal electroencephalography (EEG) without overt seizure (Chen et al., 1996, Greenberg et al., 1996). We found that *Lgl1+/−* animals have lower seizure threshold. Because our electrophysiological and biochemical results suggest higher NMDA activity in *Lgl1* mutants, we tested whether NMDA blockers could rescue any of the behavioral deficits and found that low-dose ketamine, MK801, and memantine could rescue the social interaction deficit of *Lgl+/−* animals. Therefore, apical-basal cell polarity signaling components are also essential for normal synaptic function and the loss of their function may cause neuropsychiatric disorders; these NMDA blockers may help alleviate certain behavioral symptoms of SMS patients with *Lgl1* deletion.

## Results

### Increased Glutamatergic Synapse Numbers and Reduced AMPA/NMDA Ratio in *Lgl1* Conditional Knockout *In Vivo*

**Figure 1:**
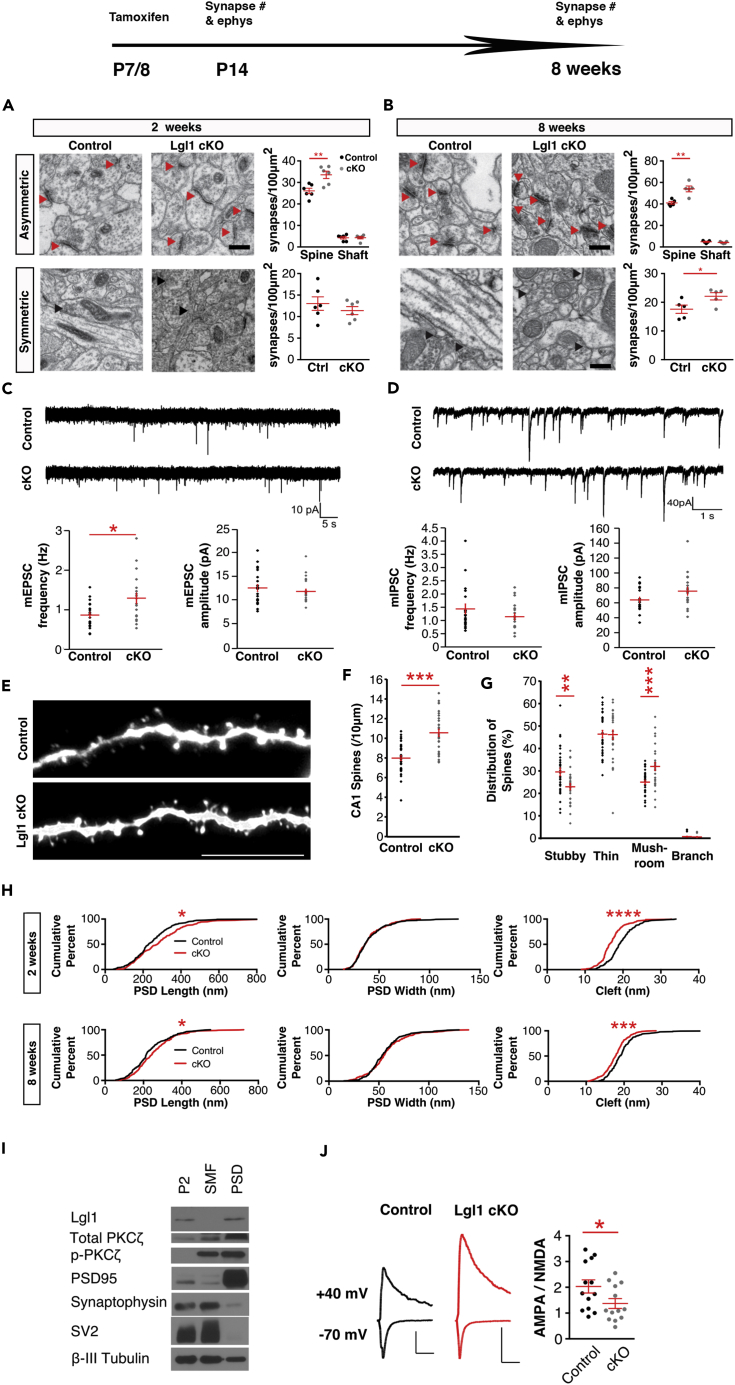
*Lgl1 Conditional Knockout* in Pyramidal Neurons Led to Increased Numbers of Asymmetric Synapses (A) Electron micrographs taken 150–200 μm (top) or 50 μm (bottom) ventral to the CA1 pyramidal neuron layer in the schaffer collateral region of P14 mice. Red arrows denote asymmetric synapses. Black arrows denote symmetric synapses. Scale bar, 500 nm. Quantification of synapse numbers corresponding to each region: N = 6 control, 6 *Lgl1* cKO animals. (B) Electron micrographs taken 150–200 μm (top) or 50 μm (bottom) ventral to the CA1 pyramidal neuron layer in the schaffer collateral region of 8-week-old mice. Red arrows denote asymmetric synapses. Black arrows denote symmetric synapses. Quantification of synapse numbers corresponding to each region: N = 5 control, 5 *Lgl1* cKO animals. (C) Representative traces of mEPSC recordings from acute slices from P13–P15 control and *Lgl1* cKO mice. Quantification of mEPSC frequency and amplitude: n = 22 control, 19 *Lgl1* cKO neurons. (D) Representative traces of mIPSC recordings from acute slices from P14 control and *Lgl1* cKO mice. Quantification of mIPSC frequency and amplitude: n = 20 control, 19 *Lgl1* cKO neurons. (E) Representative confocal images of oblique CA1 dendrites filled with Alexa Fluor 555 hydrazide. Scale bar, 10 μm. (F and G) (F) Quantification of spine density and (G) distribution of spine morphology. (H) Quantification of cumulative distributions of synapse ultrastructure measurements in P14 control and cKO animals: n = 194 control, 174 *Lgl1* cKO synapses. Quantification of cumulative distributions of synapse ultrastructure measurements in 8-week old control and cKO animals: n = 194 control, 208 *Lgl1* cKO synapses. (I) Biochemical fractionation from wild-type P14 mice. P2, crude synaptosomal; SMF, synaptic membrane fraction; PSD, postsynaptic density. (J) Representative traces of NMDAR currents and combined AMPAR/NMDAR currents from acute slices taken from P14 control and *Lgl1* cKO mice following Lgl1 deletion at P7. Scale bar, 100 pA (vertical); 100 ms (horizontal). Quantification of the calculated ratio of AMPAR current to NMDAR current: n = 13 control, 13 *Lgl1* cKO neurons. *p < 0.05; **p < 0.01; ***p < 0.001; ****p < 0.0001.

Glutamatergic synapse formation starts shortly after birth. Lgl1 has roles in earlier stages of development, including neurogenesis. To avoid early developmental defects, we conditionally knocked out *Lgl1* in hippocampal pyramidal neurons from postnatal day 7 (P7) using an inducible *Cre* line, *SLICK-H* (Figures S1A–S1C) (Heimer-McGinn and Young, 2011). Tamoxifen was injected intraperitoneally on P7 and postnatal day 8 (P8) and animals were euthanized and perfused on postnatual day 14 (P14) for electron microscopy. We counted asymmetric and symmetric synapses in the stratum radiatum. Images were taken 150–200 μm from the CA1 cell body layer in brain slices. We observed a 28.7% increase in the density of asymmetric (excitatory) synapse that are formed on dendritic spines (Figure 1A). Axo-dendritic synapses show no change in density. No significant change was observed in symmetric (inhibitory) synapse density in these slices.

To determine whether the increased synapses persist, we fixed slices from 8-week old adult mice that had tamoxifen injection at P7 and P8. In these animals, we observed an increase of 31.8% in asymmetric synapse density 150–200 μm from the CA1 cell body layer (Figure 1B). In contrast to juvenile animals, adult animals show a statistically significant 25.5% increase in density of symmetric synapses in the region 50 μm from the CA1 cell layer. The delay in the increase of symmetric synapses suggests that it is not directly caused by the loss of function of *Lgl1*, but possibly by a homeostatic response of the hippocampal circuitry. The number of asymmetric synapses in the region 50 μm from the CA1 cell layer also shows an increase (Figures S1D and S1E).

To assess the function of the increased synapses, we recorded miniature excitatory postsynaptic currents (mEPSCs) and miniature inhibitory postsynaptic currents (mIPSCs) from acute brain slices from P14 and P15 control and *Lgl1 cKO* animals. Quantification of frequency and amplitude of synaptic currents indicates that mEPSC frequency was increased by 38%, whereas amplitude was not changed significantly, indicating an increase in synapse number but similar AMPA-R composition (Figure 1C). No significant changes were observed in mIPSC currents (Figure 1D), consistent with our electron microscopy data. Consistent with this, cultured hippocampal neurons from mice carrying germline deletion of *Lgl1* also showed increased colocalization between PSD95 and vGlut1 puncta at 14 days *in vitro* (Figures S1F and S1G). mEPSC kinetics did not show significant changes (Figure S1H), whereas mIPSC kinetics only showed a significant decrease in decay time constant, but not in other measures (Figure S1I).

To determine whether dendritic spine density was affected, we filled neurons from fixed brain sections with Alexa 555 dye to visualize spines in yellow fluorescent protein (YFP)-positive CA1 pyramidal neurons (Figure 1E). We found that overall spine density was indeed increased by 38% in *Lgl1* cKO mice (Figure 1F). We also characterized the morphology of spines at P14. Compared with control, *Lgl1* cKO mice showed more mushroom spines and a reduced proportion of stubby spines (Figure 1G). As mushroom spines represent stabilized synapses and thin spines are unstable, this suggests that *Lgl1* cKO led to functionally hyperconnected circuits. Consistent with this, we then quantified the ultrastructure from the electron micrograph and found that the length of postsynaptic density (PSD) was increased and the gap of synaptic cleft was reduced at P14 and at age 8 weeks (Figure 1H). As Lgl1 interacts with the MAGUK proteins, we determined the subcellular localization and found that Lgl1 was present in the postsynaptic density (Figure 1I). We also measured AMPA/NMDA ratio, and found that the ratio is decreased in the *Lgl1 cKO*, suggesting altered glutamate receptor trafficking or function (Figure 1J).

### Lgl1 Negatively Regulates Synapse Number by Inhibiting the Atypical PKCs

As Lgl1 and aPKC antagonize each other in cell polarity signaling, we asked whether Lgl1 may regulate synapse formation by inhibiting the aPKCs. To validate whether Lgl1 also inhibits the aPKCs in neurons, we tested their interaction in neural progenitor cells from E11.5 mouse telencephalon. *Lgl1* cKO and control cells were generated by treatment of cultures with AD5-CMV-Cre and Ad5-CMV-GFP adenoviruses (Vector Development Laboratory, Baylor College of Medicine), respectively, and verified that Lgl1 protein was completely lost in the cKO (Figure S2A). We then tested whether aPKC activity, as evident by association with Par3, was increased in *Lgl1* cKO. Activated aPKC (phosphorylated at T555) and aPKC interaction with Par3 were found strongly increased in *Lgl1* cKO, confirming the increase of apical signaling and decrease of basal-lateral signaling.

**Figure 2:**
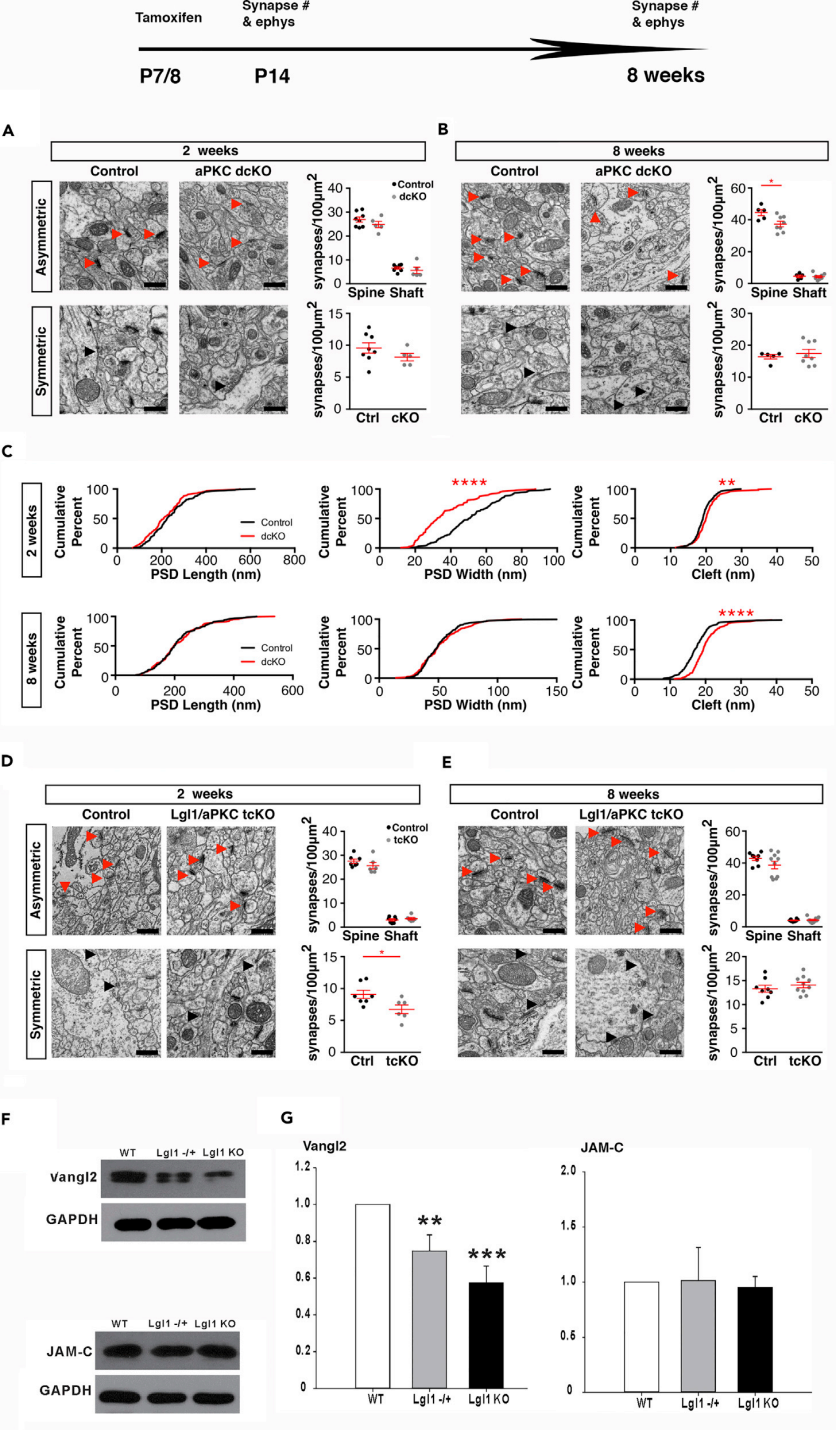
Lgl1 Inhibits Synapse Formation by Inhibiting aPKC and Promotes the Expression of Vangl2 (A) Electron micrographs taken 150–200 μm (top) or 50 μm (bottom) ventral to the CA1 pyramidal neuron layer in the schaffer collateral region of P14 mice. Red arrows denote asymmetric synapses. Black arrows denote symmetric synapses. Scale bar, 500 nm. Quantification of synapse numbers corresponding to each region: N = 8 control, 5 *aPKC dcKO* animals. (B) Electron micrographs taken 150–200 μm (top) or 50 μm (bottom) ventral to the CA1 pyramidal neuron layer in the schaffer collateral region of 8-week-old mice. Red arrows denote asymmetric synapses. Black arrows denote symmetric synapses. Quantification of synapse numbers corresponding to each region: N = 5 control, 8 *aPKC dcKO* animals. (C) Quantification of cumulative distribution of synapse ultrastructure measurements in P14 control and cKO animals: n = 194 control, 129 *aPKC dcKO* synapses. Quantification of cumulative distribution of synapse ultrastructure measurements in 8-week-old control and cKO animals: n = 87 control, 168 *aPKC dcKO* synapses. (D) Electron micrographs taken 150–200 μm (top) or 50 μm (bottom) ventral to the CA1 pyramidal neuron layer in the schaffer collateral region of P14 mice. Red arrows denote asymmetric synapses. Black arrows denote symmetric synapses. Scale bar, 500 nm. Quantification of synapse numbers corresponding to each region: N = 7 control, 6 *Lgl1;PKCι/λ;PKCζ tcKO* animals. (E) Electron micrographs taken 150–200 μm (top) or 50 μm (bottom) ventral to the CA1 pyramidal neuron layer in the schaffer collateral region of 8-week-old mice. Red arrows denote asymmetric synapses. Black arrows denote symmetric synapses. Quantification of synapse numbers corresponding to each region: N = 8 control, 10 *Lgl1;PKCι/λ;PKCζ tcKO* animals. (F) Levels of Vangl2 and JAM-C proteins in P2 fractions by Western blots. (G) Quantification of Vangl2 and JAM-C protein levels in P2 fraction. N = 5 for Vangl2. N = 4 for JAM-C. *p < 0.05; **p < 0.01; ***p < 0.001; ****p < 0.0001.

There are two isoforms of aPKCs in mouse. We used the same strategy of tamoxifen-induced deletion of *aPKC*s using *SLICK-H* to delete both isoforms of *aPKCs, PKCι/λ* and *PKCζ,* to eliminate the possibility of compensation. Following this deletion, we counted asymmetric and symmetric synapses from the schaffer collateral 150 μm from the CA1 cell layer of 2- and 8-week-old animals. At 2 weeks, we observed no significant difference in the number of asymmetric or symmetric synapses (Figure 2A). However, at 8 weeks, there was a significant decrease (−16.1%) in the number of asymmetric synapses (Figure 2B). In the proximal region 50 μm from the CA1 cell body layer, we also observed a significant decrease at 8 weeks, but not at 2 weeks (Figures S2B and S2C). Therefore, aPKC is not essential for initial glutamatergic synapse formation but required for their stability and maintenance. We then analyzed the ultrastructure and found that the PSD width was reduced at 2 weeks and the synaptic cleft was increased at 2 weeks and that the magnitude of this change increased at 8 weeks (Figure 2C). This suggests that aPKC is likely important for the stability of synapses, the opposite of Lgl1 (Figure 1H).

We then asked whether simultaneous deletion of *Lgl1*, *PKCι/λ,* and *PKCζ* might lead to mitigation of the effects observed in the *Lgl11* conditional deletion experiments. In 2-week-old *Lgl1*:*PKCι/λ*:*PKCζ* tcKO animals, we observe no significant change in asymmetric synapse number, suggesting that the increase of synapse numbers in *Lgl1 cKO* may be partly due to the increase of aPKC activity (Figure 2D). However, we observed a significant decrease (−25.9%) in the number of symmetric synapses. In 8-week-old *Lgl1*:*PKCι/λ*:*PKCζ* tcKO animals, we observed no significant change in asymmetric or symmetric synapse number (Figure 2E). No significant difference in asymmetric synapse density was observed in the proximal region at 2 and 8 weeks (Figures S3A and S3B). Therefore, Lgl1 may negatively regulate glutamatergic synapse numbers by inhibiting aPKC, which is required for the stability and maintenance of glutamatergic synapses.

Because PCP proteins regulate synapse formation and apical-basal polarity signaling regulates the localization PCP signaling components, we tested whether Lgl1 may regulate PCP components using synaptosome fractionation. The SLICK-H line (inducible Cre) expresses Cre in only 60% of pyramidal neurons at P7. Therefore, we cultured neurons from *Lgl1* KOs, heterozygotes, and wild-type and extracted synaptosome fraction. We found that Vangl2 protein levels are decreased in the P2 fractions of *Lgl1+/−* and *Lgl1−/−* compared with that of the *wild-type* (Figure 2F), whereas the levels of an adhesion molecule Jam-C were not affected (Figure 2G). This is consistent with the inhibitory function of Vangl2 in glutamatergic synapse formation.

### Lgl1 Controls Glutamatergic Synapse Number and Is Required for Synaptic Plasticity in Adulthood

**Figure 3:**
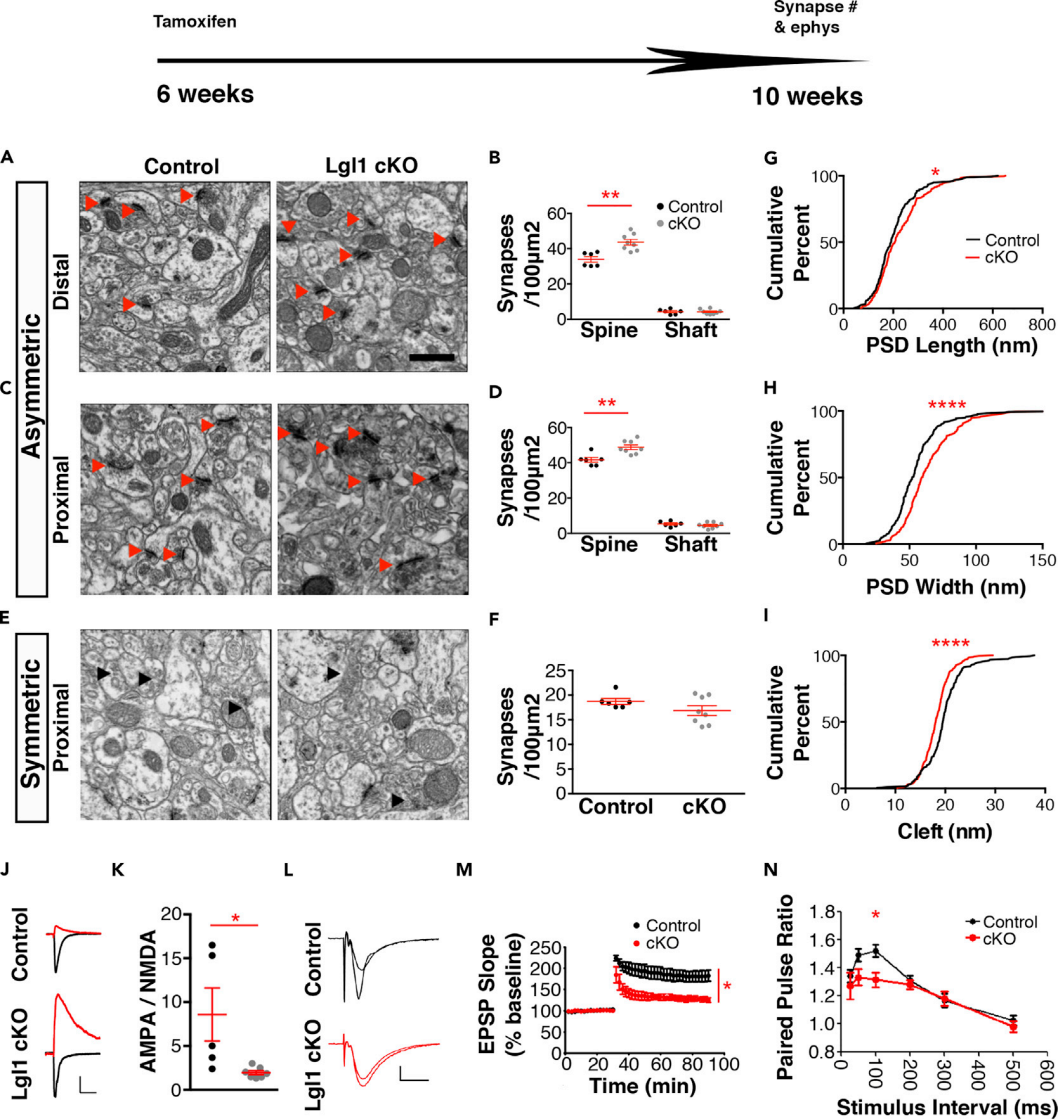
Increased Synapse Numbers, Altered AMPA/NMDA Ratio, and Impaired Plasticity in Adult Conditional Knockout of *Lgl1* (A) Electron micrographs of the schaffer collateral (SC) 150–200 μm ventral to the CA1 pyramidal cell layer of slices from 10-week-old control and *Lgl1* cKO mice following deletion of Lgl1 beginning at 6 weeks of age. Red arrows denote asymmetric synapses. Scale bar, 500 nm. N = 6 control, 8 *Lgl1* cKO animals. (B) Quantification of asymmetric synapse density of the region described in Figure 5A. (C) Electron micrographs of the SC 50 μm ventral to the CA1 pyramidal cell layer in 10-week-old animals. (D) Quantification of asymmetric synapses in the proximal region. (E) Micrographs showing symmetric synapses (black arrows) in the proximal region of the SC. (F) Quantification of symmetric synapses. (G) Quantification of cumulative frequency for postsynaptic density (PSD) length. (H) Quantification for PSD width. (I) Quantification of synaptic cleft distance. n = 180 *Lgl1* control synapses, 242 *Lgl1* cKO synapses. (J) Representative traces of NMDAR current and combined AMPAR/NMDAR current from acute slices taken from 6-week-old control and *Lgl1* cKO mice following Lgl1 deletion beginning at P28. Scale bar, 50 pA, 80 ms. (K) Quantification of the calculated ratio of AMPAR to NMDAR current. N = 5 control, 7 *Lgl1* cKO neurons. (L) Representative traces of EPSPs before and after TBS stimulation was delivered to acute slices from control and *Lgl1* cKO mice. Scale bar, 0.2 mV, 10 ms. (M) Quantification of EPSP slope before and after theta burst stimulation (TBS). N = 5 *Lgl1* control, 4 *Lgl1* cKO. (N) Quantification of paired-pulse ratio from control and *Lgl1* cKO animals deleted at 6 weeks. N = 6 *Lgl1* control, 6 *Lgl1* cKO. *p < 0.05; **p < 0.01; ****p < 0.0001.

Because *Lgl1* is highly expressed in the adult central nervous system, including the hippocampus, we next characterized the role of Lgl1 in the adult brain. By early adulthood at age 6 weeks, synapse formation has slowed considerably in the rodent hippocampus compared with postnatal development (Wang et al., 2007). We conditionally knocked out *Lgl1* by injecting tamoxifen at 6 weeks after birth in SLICK-H animals. We then used electron microscopy to assess the density of asymmetric and symmetric synapses in the schaffer collateral in 10-week-old animals. Asymmetric synapse density in the region 150–200 μm distal to the CA1 cell body layer was again increased by 28.6% on the dendritic spines (Figures 3A and 3B). A similar increase was observed in the proximal region 50 μm from the CA1 cell layer (Figures 3C and 3D). Symmetric synapses were again unaffected (Figures 3E and 3F). In addition, synapse ultrastructure was altered in the adult deletion of *Lgl1*, with longer and wider PSDs (Figures 3G and 3H) and smaller synaptic clefts (Figure 3I), possibly as a result of altered biochemical makeup of synapses. We performed patch clamping with P42 slices to assess the synaptic receptor expression from animals with *Lgl1* deleted beginning at P28. We observed a severe reduction in the AMPA/NMDA ratio in neurons from *Lgl1* cKO mice (Figures 3J and 3K), much greater than was observed at P14 following deletion of *Lgl1* at P7/P8. These results suggest that *Lgl1* is also required for control of synapse number and quality in adulthood.

We tested synaptic plasticity using hippocampal slices from 10-week old mice following conditional *Lgl1* deletion at age 6 weeks. Slices from *Lgl1* cKO animals showed impaired long-term potentiation induction in response to theta burst stimulation (TBS) (Figures 3L–3M). In addition, slices from *Lgl1* cKO mice showed impaired paired-pulse facilitation when stimuli were separated by 100 ms (Figure 3N), indicating altered synaptic release.

### *Lgl1* cKO Mice Showed Behavioral Deficits

**Figure 4:**
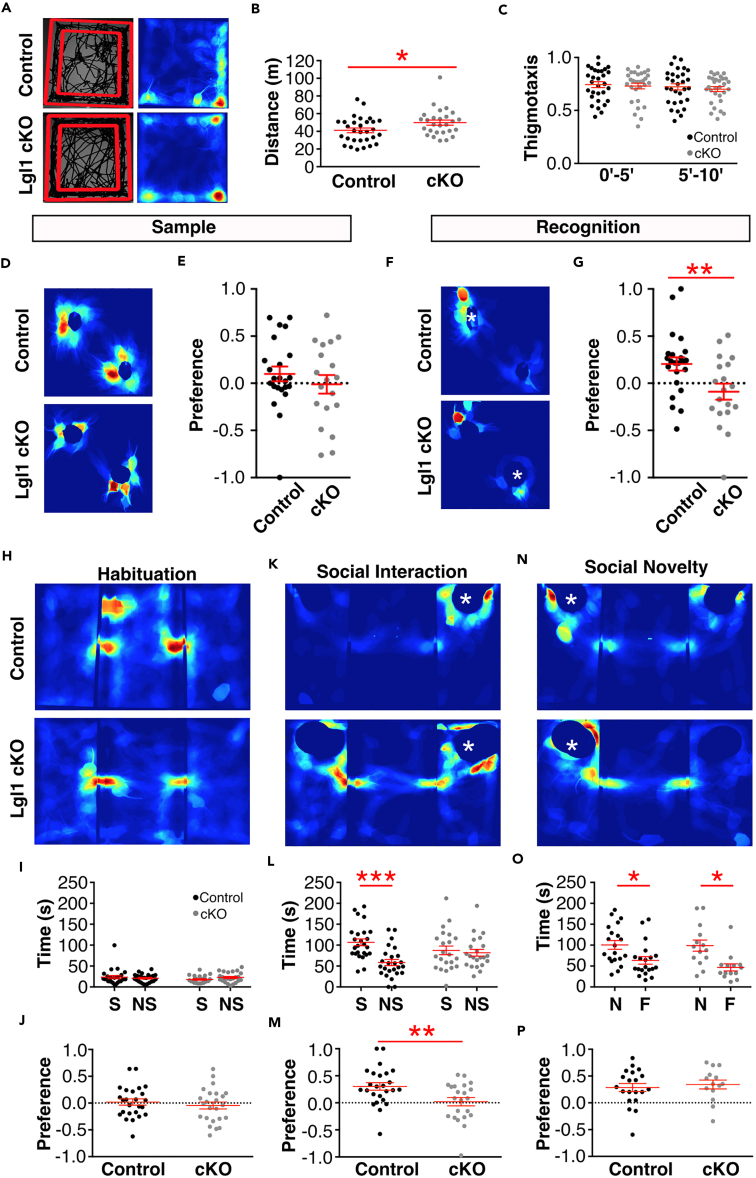
*Lgl1* Deletion at P7 Produced a Subset of SMS-like Behavioral Phenotypes (A) Open-field analysis following P7 deletion of Lgl1 showing representative trajectories (left) and heatmaps (right) of control and *Lgl1* cKO animals. (B) Quantification of distance traveled during the test. N = 30 control, 27 *Lgl1* cKO. (C) Quantification of time spent in the outer region of the field (thigmotaxis) during the first and second 5-min periods. (D and E) (D) Representative heatmap and quantification (E) of animal preference for objects during the sample phase of the novel object recognition (NOR) test. N = 23 control, 19 *Lgl1* cKO animals. (F and G) (F) Representative heatmaps and quantification (G) of animal preference for objects during the test phase of the NOR test. White asterisk denotes location of the novel object. (H) Representative heatmaps from the habituation period of the social interaction (SI) test. (I and J) (I) Quantification of time spent in and (J) preference for regions of interest (ROIs) representing future location of novel mice and objects. N = 25 control, 24 *Lgl1* cKO animals. (K) Representative heatmaps during the social interaction phase of the SI test. White asterisk denotes location of the novel mouse. Opposite chamber contains the object. (L and M) (L) Quantification of time spent in and (M) preference for ROIs containing either the novel mouse or novel object. Positive value indicates preference for the novel mouse. N = 25 control, 22 *Lgl1* cKO animals. (N) Representative heatmaps during the social novelty phase of the SI test. White asterisk denotes location of the novel mouse. Opposite chamber contains the previously explored mouse. (O and P) (O) Quantification of time spent interacting with and (P) preference for mice during the social novelty phase. Positive value indicates preference for the novel mouse. N = 20 control, 14 *Lgl1* cKO animals. NS, nonsocial; S, social. ∗p < 0.05; ∗∗p < 0.01; ∗∗∗p < 0.001.

*Lgl1* is frequently deleted in SMS. But the genes responsible for the behavioral symptoms of SMS have not been well understood. To test whether deletion of *Lgl1* in pyramidal neurons may contribute to the behavioral deficits, we performed a number of behavioral tests. We assessed locomotor activity and exploratory behavior using an open field test (Figure 4A; Gould et al., 2009). *Lgl1 cKO* animals showed increased locomotor activity, traveling 20% further during the 10-min test than control mice (Figure 4B). No significant changes were observed in thigmotaxis, the preference for the outside of the field versus the center region (Figure 4C). Animals did not show a difference in the amount of time spent self-grooming during the open-field test (Figure S4A), but showed a significant increase in rearing activity, an exploratory behavior (Figure S4B).

To assess cognitive function following conditional *Lgl1* deletion, we tested the *Lgl1 cKO* crossed with SLICK-H in the novel object recognition (NOR) paradigm. The NOR test assesses whether an animal can distinguish a novel object from a previously explored familiar object. We calculated a preference index from each animal by subtracting the familiar object interaction time from the novel exploration time and normalizing to the total exploration time ((novel-familiar)/(novel + familiar)). During the sample phase, when both objects were novel, animals showed no preference and would randomly explore both objects (Figures 4D and 4E). When a novel object was introduced after a 2-min delay, control animals showed a strong preference for the novel object, whereas *Lgl1* cKO animals continued to explore randomly and maintain a preference index close to zero (Figures 4F and 4G).

Brain hyperconnectivity has been associated with ASDs (Dominguez et al., 2013, Keown et al., 2013, Supekar et al., 2013). Sociability in the three-chamber social interaction task has been used extensively to assess social behavior in mice (Yang et al., 2011). We tested *Lgl1 cKO* mice crossed with SLICK-H to assess their sociability (Figures 4H–4P). Before introduction of the target mouse, animals showed no preference for either side, exploring the field randomly (Figures 4H–4J). When a mouse was introduced to one side of the field, whereas an empty enclosure was introduced to the opposite side, control animals showed a strong preference for interacting with the novel mouse. *Lgl1 cKO* animals showed no preference (Figures 4K–4M) and spent similar amount of time exploring the novel mouse and novel object. Interestingly, no statistically significant difference was observed in the final phase of the test where subject mice were given a choice between a familiar and novel target mouse (Figures 4N–4P). Similar to controls, *Lgl1 cKO* mice still appeared to show a preference for the novel mouse. *Lgl1 cKO* mice showed normal spatial memory by alternations and entries in the Y-maze (Figures S4C and S4D) and normal visual performance (Figure S4E). Hippocampal- and amygdala-dependent memory formation was spared in the conditioned fear task (Figure S4F). Nestlet-shredding activity was unchanged in the conditional deletion of *Lgl1* (Figure S4G).

### *Atypical PKC* Deletion Partially Rescued Behavioral Deficits of *Lgl1 cKO*

**Figure 5:**
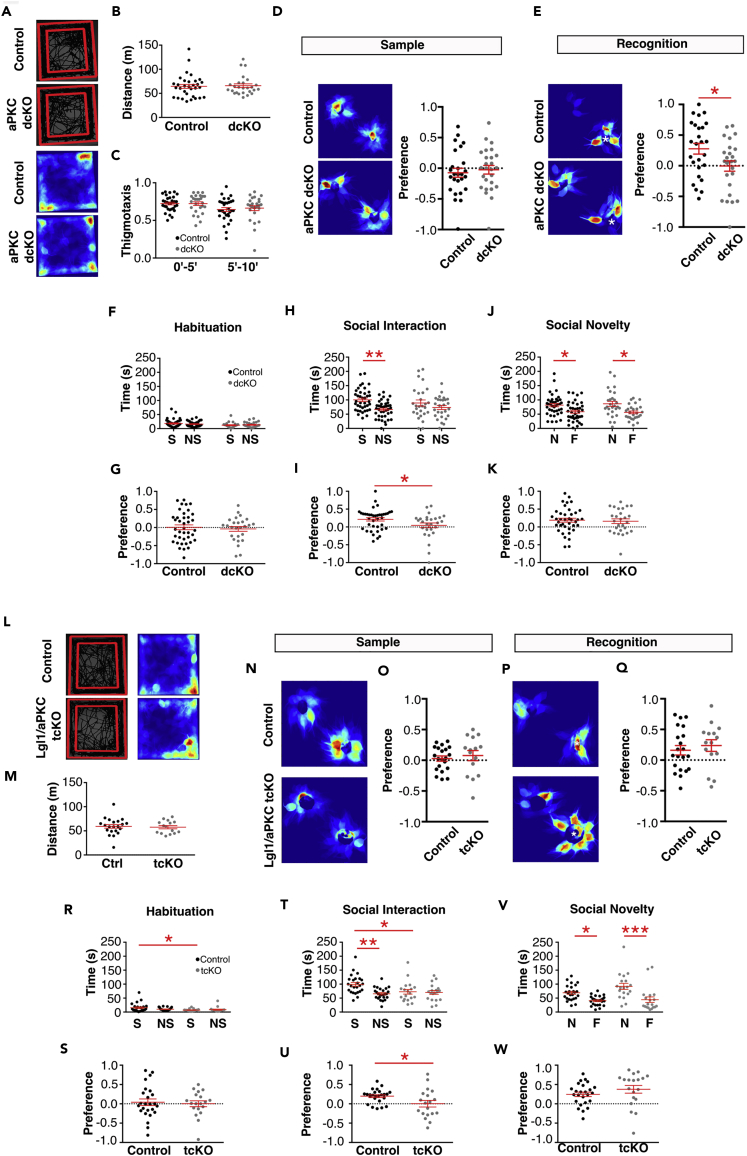
Conditional Triple Knockout of Lgl1, PKCι/λ, and PKCζ Rescued Asymmetric Synapse Number and Cognitive Deficit (A) Open-field analysis following P7 deletion of Lgl1 showing representative trajectories (left) and heatmaps (right) of control and *aPKC dcKO* animals. (B) Quantification of distance traveled during the test. N = 32 control, 26 *aPKC dcKO* animals. (C) Quantification of time spent in the outer region of the field (thigmotaxis) during the first and second 5-min periods. (D) Representative heatmap and quantification of animal preference for objects during the sample phase of the novel object recognition (NOR) test. N = 26 control, 15 *aPKC dcKO* animals. (E) Representative heatmaps and quantification of animal preference for objects during the test phase of the NOR test. White asterisk denotes location of the novel object. (F and G) (F) Quantification of time spent in and (G) preference for regions of interest (ROIs) representing future location of novel mice and objects. N = 35 control, 26 *aPKC dcKO* animals. (H and I) (H) Quantification of time spent in and (I) preference for ROIs containing either the novel mouse or novel object. Positive value indicates preference for the novel mouse. N = 35 control, 27 *aPKC dcKO* animals. *p < 0.05; **p < 0.01. (J and K) (J) Quantification of time spent interacting with and (K) preference for target mice during the social novelty phase. Positive value indicates preference for the novel mouse. N = 35 control, 27 *aPKC dcKO* animals. *p < 0.05. (L) Open-field analysis following P7 deletion showing representative trajectories (left) and heatmaps (right) of control and *Lgl1;PKCι/λ;PKCζ tcKO* animals. (M) Quantification of distance traveled during the test. N = 20 control, 14 *Lgl1;PKCι/λ;PKCζ tcKO* animals. (N and O) (N) Representative heatmap and quantification (O) of animal preference for objects during the sample phase of the novel object recognition (NOR) test. N = 21 control, 15 *Lgl1;PKCι/λ;PKCζ tcKO* animals. (P and Q) (P) Representative heatmaps and quantification (Q) of animal preference for objects during the recognition test phase of the NOR test. White asterisk denotes location of the novel object. (R and S) (R) Quantification of time spent in and (S) preference for ROIs representing future location of novel mice and objects. (T and U) (T) Quantification of time spent in and (U) preference for ROIs containing either the novel mouse or novel object. Positive value indicates preference for the novel mouse. (V and W) (V) Quantification of time spent interacting with and (W) preference for target mice during the social novelty phase. Positive value indicates preference for the novel mouse. N = 25 control, 19 *Lgl1;PKCι/λ;PKCζ tcKO* animals. NS, nonsocial; S, social. *p < 0.05; **p < 0.01; ***p < 0.001.

Having observed synapse phenotypes in the conditional *aPKC* deletion, we then tested whether the changes would lead to behavioral deficits. In open-field test, *PKCι/λ* and *PKCζ dcKO* animals showed no significant changes in locomotor activity or thigmotaxis (Figures 5A–5C). We also assessed cognitive function and found that *PKCι/λ*:*PKCζ dcKO* were impaired in the novel object recognition test (Figures 5D and 5E). In the social interaction test, animals showed no preference before the introduction of the novel mouse (Figures 5F and 5G). Littermate control animals preferred social interaction, spending more time with the novel mouse versus the novel object, whereas *PKCι/λ*:*PKCζ dcKO* spent similar amounts of time interacting with the novel mouse and novel object, maintaining a preference index close to 0 (Figures 5H and 5I). Preference for social novelty was unaffected by aPKC deletion (Figures 5J and 5K).

We then tested locomotor activity and observed no difference between littermate control and *Lgl1*:*PKCι/λ*:*PKCζ* tcKO animals (Figures 5L–5M). However, triple conditional deletion of *Lgl1*, *PKCι/λ,* and *PKCζ* did rescue cognitive deficit in the novel object recognition test, with *Lgl1*:*PKCι/λ*:*PKCζ* tcKO animals performing similarly to littermate controls and better than chance (one-sample t test, p = 0.0262) in the test (Figures 5N–5Q). Our observations showed that conditional deletion of *Lgl1*, *PKCι/λ*, and *PKCζ* corrects synapse density changes observed and preserves cognitive function. In the social interaction test (Figures 5R–5W), tcKO animals still showed impairment in the social interaction phase (Figures 5T–5U), but not the social novelty phase (Figures 5V–5W).

### *Lgl1* Heterozygotes Had Increased Synapse Numbers and Displayed Behavioral Deficits Suggesting a Role in Smith-Magenis Syndrome

**Figure 6:**
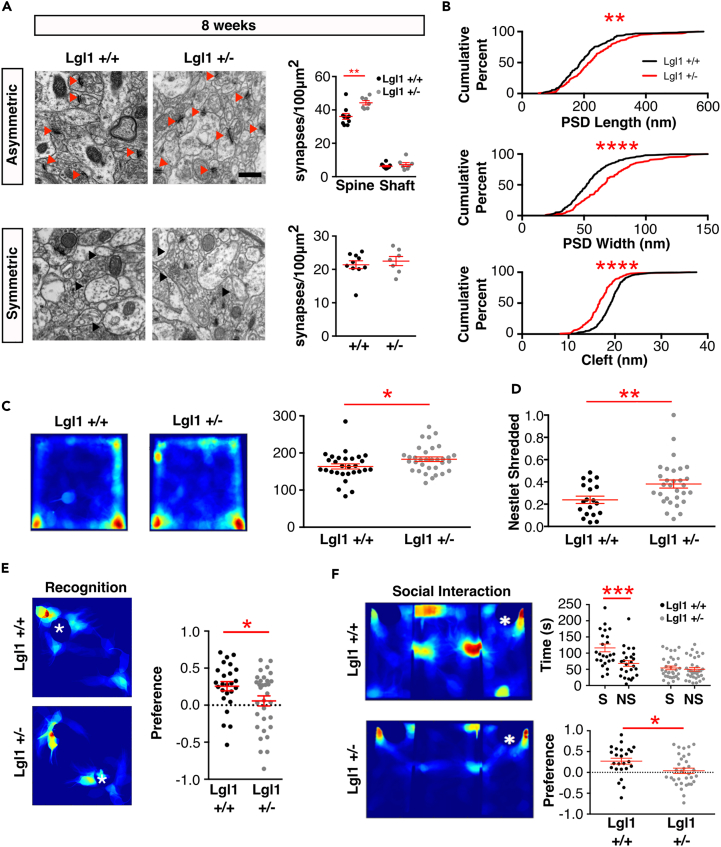
*Lgl1*+/− Mice Showed Increased Synaptic Numbers and SMS-like Behavioral Phenotypes (A) Electron micrographs taken 150–200 μm (top) or 50 μm (bottom) ventral to the CA1 pyramidal neuron layer in the schaffer collateral region of 8-week-old mice. Red arrows denote asymmetric synapses. Black arrows denote symmetric synapses. Scale bar, 500 nm. Quantification of synapse numbers corresponding to each region: N = 10 *Lgl1*+/−, 7 *Lgl1*+/− animals. (B) Quantification of cumulative distribution of synapse ultrastructure measurements in 8-week old *Lgl1*+/+ and *Lgl1*+/− animals: n = 265 *Lgl1*+/+ synapses, 140 *Lgl1*+/− synapses. (C) Representative heatmaps from the 1-h extended-duration open-field test for *Lgl1*+/+ and *Lgl1*+/− animals and quantification for distance traveled in the extended open-field test. N = 30 *Lgl1*+/+, 33 *Lgl1*+/−. (D) Quantification of nestlet-shredding activity. N = 19 *Lgl1*+/+, 30 *Lgl1*+/− animals. (E and F) (E) Representative heatmaps from the recognition test phase of the novel object recognition (NOR) test 24 h after the sample phase of the object. Quantification of object preference during the test phase 24 h after the sample phase. N = 25 *Lgl1*+/+, 32 *Lgl1*+/−. (F) Representative heatmaps during the social interaction phase of the social interaction (SI) test. White asterisk denotes location of the novel mouse. Quantification of interaction preference in the SI test. N = 25 *Lgl1*+/+, 33 *Lgl1*+/− animals. ∗p < 0.05; ∗∗p < 0.01; ∗∗∗p < 0.001; ∗∗∗∗p < 0.0001.

As SMS arises from heterozygous deletion of the critical region, we assessed the effects of germline heterozygous deletion of *Lgl1* in our mouse (Klezovitch et al., 2004). We imaged hippocampal sections from 8-week-old control and *Lgl1* heterozygous animals from the germline *Lgl1* KO line using electron microscopy. In these animals, the region 150–200 μm from the CA1 cell body layer showed an increase in asymmetric synapse density, with a somewhat smaller but significant increase (22.6%; Figure 6A and proximal region: S5A) compared with what was observed when both copies were deleted in the conditional KO. Symmetric synapses were not affected. Analysis of synapse ultrastructure revealed longer and wider PSDs and smaller synaptic clefts (Figure 6B).

In the open-field test, *Lgl1*+/− animals did not show a significant difference from control animals after 10 min (Figures S5B and S5C). During an extended observation period of 60 min in the open field (Figure 6C), *Lgl1*^*+/−*^ animals showed increased locomotor activity by 12.0% overall.

We also tested stereotyped repetitive behavior and found that *Lgl1*+/− animals showed increased nestlet shredding when given cotton nesting material in a novel cage (Figure 6D), shredding 59.4% more material during the test. As this behavior was not observed in *Lgl1* cKO, this is likely a result of *Lgl1* deletion in heterozygotes in the subcortical areas that are not affected by the *Thy-1*-dependent deletion in the *Lgl1* cKO animals crossed with SLICK-H.

Preference for novel object recognition was spared in *Lgl*+/− animals (Figures S5E–S5H) following a 2-min delay, suggesting that this cognitive task might be partially spared by either reduced impact on synapse density or synaptic function due to the remaining copy of *Lgl1*. Therefore, we performed an additional novel object recognition test, this time with a 24-h delay between the initial sample period and the recognition test. After 24 h, control animals successfully discriminated novel and familiar objects, whereas *Lgl1*+/− animals did not (Figure 6E). Patients with SMS demonstrate mild to moderate cognitive impairment or developmental delay, and it is likely that the partially preserved recognition of novel objects reflects a mild cognitive impairment in the mouse model.

Similar to *Lgl1* cKO animals, *Lgl1*+/− animals demonstrated deficient social interaction, but spared preference for social novelty (Figures 6F, S5I, and S5J), indicating that loss of one copy of *Lgl1—*as occurs in SMS*—*may be sufficient to give rise to ASD-like behaviors. *Lgl1*+/− animals did not show a difference from control littermates in age-dependent weight gain (Figure S5K).

### Social Interaction Deficit in *Lgl1+/−* May Be Caused by Excessive NMDA Current

**Figure 7:**
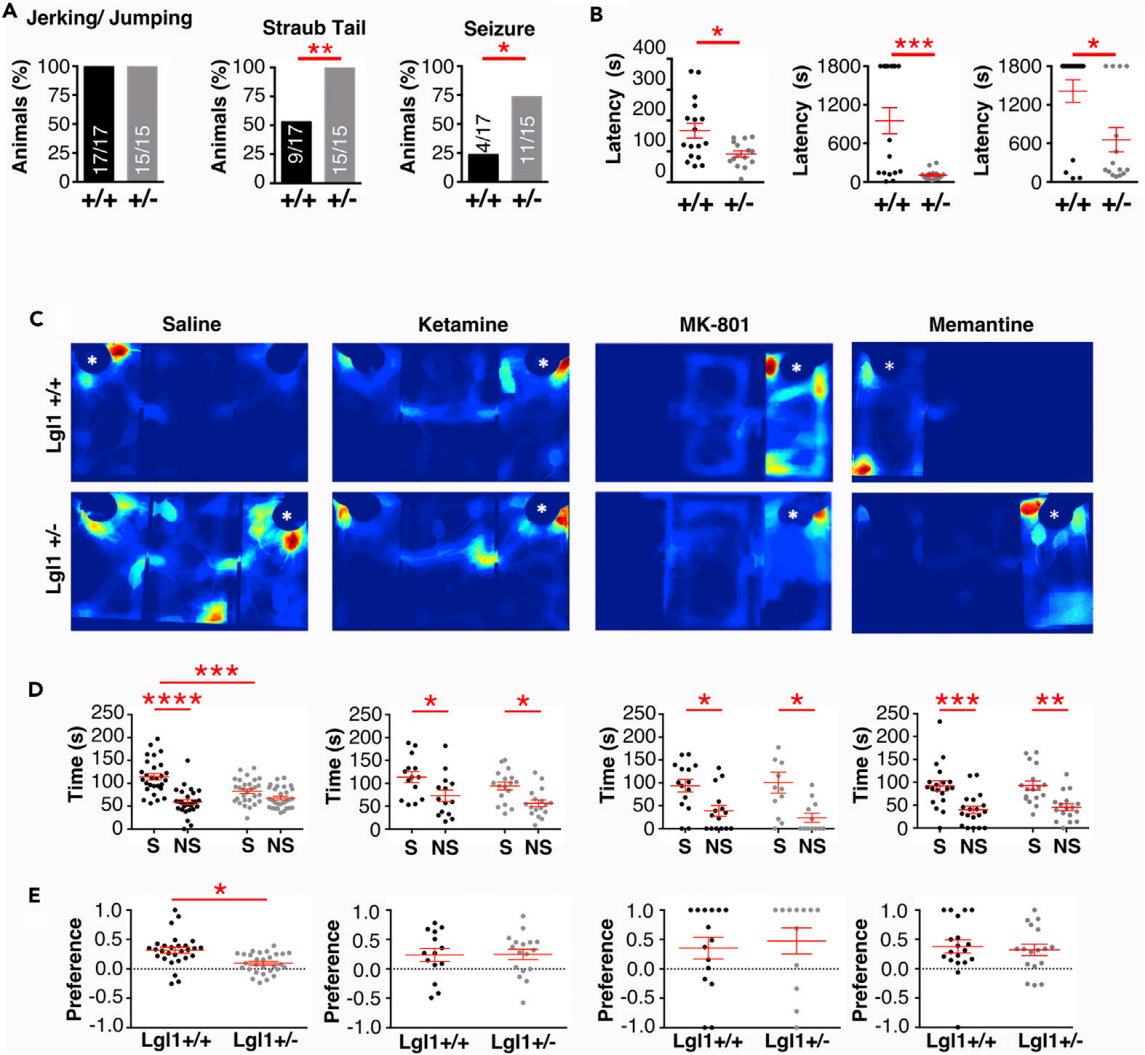
Decreased Seizure Threshold in *Lgl1*+/− Mice and Rescue of Social Interaction by NMDAR Blockade (A) Quantification of the occurrence of jerking/jumping, Straub tail, and clonic-tonic seizures in *Lgl1*+/+ and *Lgl1*+/− animals following 50 mg/kg PTZ administration. Fisher's exact test. (B) Quantification of the latency to the first observation of behaviors following 50 mg/kg PTZ administration. N = 17 *Lgl1*+/+, 15 *Lgl1*+/− animals. Mann-Whitney U statistic test. (C) Representative heatmaps during the social interaction phase of the social interaction test following intraperitoneal injection with 50 μL saline, 30 mg/kg ketamine, 0.3 mg/kg MK-801, or 20 mg/kg memantine. White asterisk denotes location of the novel mouse. (D and E) (D) Quantification of time spent in social (S) or nonsocial (NS) regions of interest and (E) interaction preference of treated animals. N = (saline) 17 *Lgl1*+/+, 19 *Lgl1*+/− animals; (ketamine) 14 *Lgl1*+/+, 17 *Lgl1*+/− animals; (MK-801) 13 *Lgl1*+/+, 14 *Lgl1*+/− animals; (memantine) 19 *Lgl1*+/+, 17 *Lgl1*+/− animals. *p < 0.05; **p < 0.01; ***p < 0.001; ****p < 0.0001.

Because SMS often presents with either seizures or abnormal EEG without overt seizure (Chen et al., 1996, Greenberg et al., 1996), we tested whether abnormally high synapse density and NMDA current would lower the seizure threshold in response to the GABA_A_-blocking drug pentylenetetrazol (PTZ). Following injection of 50 mg/kg PTZ, the occurrence and latency of activities indicating the onset of a seizure were recorded. All animals tested showed at least one instance of jumping or full-body jerking (Figure 7A), whereas *Lgl1+/−* animals showed a shorter latency to the behavior following PTZ administration (Figure 7B). *Lgl1+/−* animals also showed significantly higher occurrence and shorter latency to Straub Tail, indicating persistent muscle contraction, and also showed a similar effect for the occurrence of clonic-tonic seizures.

The lower AMPA/NMDA ratio and unaffected AMPA current suggests that *Lgl1* mutants may have excessive NMDA current. We asked whether abnormal NMDAR-dependent signaling could underlie some of the behavioral deficit, especially social interaction, that we observed. We used pharmacological blockers of NMDARs and tested animals in the three-chamber social interaction test. Subanesthetic dose of ketamine has been shown to provide rapid blockage of NMDAR-dependent signaling. We tested social interaction with injection of 50 μL sterile saline and observed that control animals preferred social interaction, whereas *Lgl1*+/− mice did not show a preference (Figures 7C–7E). Following injection of 30 mg/kg ketamine, *Lgl1*+/− mice showed a clear preference for social interaction similar to what was observed from control animals (Figures 7C–7E). Preferences in habituation and social novelty phases are unaffected by saline or drug injection (Figures S6A–S6D). Ketamine injection did not change nestlet-shredding activity (Figure S6E). In addition, we also tested MK-801 (dizocilpine) at 0.3 mg/kg and memantine at 20 mg/kg and observed that *Lgl1*+/− strongly preferred social interaction, as did control mice (Figures 7C–7E). These results suggest that excessive NMDA current due to *Lgl1* deletion may contribute to some of the behavioral deficits and that inhibiting NMDARs may help alleviate some of the neuropsychiatric symptoms in SMS patients with *Lgl1* deletion.

## Discussion

Although many of the proteins in glutamatergic synapses have been identified and their roles in synapse formation and function have been studied, the signaling logic that orchestrates the assembly of hundreds of proteins into a highly organized and dynamic structure remains poorly known. We show here that a conserved apical-basal polarity signaling component, Lgl1, is localized in the PSD and regulates synapse numbers and compositions of key synaptic proteins and glutamate receptors, probably by inhibiting aPKC activity and via interactions with its conserved binding partners, particularly the MAGUKs. Atypical PKC has been studied for its role in memory formation and consolidation. Here we report that conditional deletion of both isoforms of aPKCs at P7 and P8 led to a reduction in synapse number in adulthood and cognitive and social deficits. The observation that deletion of Lgl1 or aPKC both lead to behavioral changes indicates that synapse number must be optimally controlled for behavioral functions and changes in either direction lead to impairment. Triple conditional knockout of Lgl1 and aPKC isoforms rescued the number of the asymmetric synapses and cognitive function, supporting their antagonistic functions in synapse formation. Lgl1 forms a basal complex with Discs Large, which is the homolog of MAGUKs, essential scaffold proteins in the postsynaptic density, which regulate trafficking and clustering of glutamate receptors. Therefore, loss of Lgl1 may lead to changes of MAGUKs and glutamate receptor compositions, such as the reduction of AMPA/NMDA ratio. It should be noted that we also observed a decrease of paired-pulse ratio in *Lgl1 cKO*, suggesting that there may also be presynaptic defects (Figure 3N). The Cre line we used here, *SLICK-H*, expresses CreERT2 in both CA3 and CA1 pyramidal neurons. Although Lgl1 was found to be present in the postsynaptic density (Figure 1I), Lgl1 may also have a function on the presynaptic side. This reduction of paired-pulse ratio may contribute to the altered synaptic function.

*Lgl1* is frequently deleted in SMS; therefore, we performed a number of behavioral tests. cKO of *Lg11* from P7 and P8 led to behavioral deficits, including hyperactivity, cognitive impairment, and social interaction, consistent with the autism-like symptoms in SMS. As SMS involves the microdeletion of one of the chromosomes, we analyzed the *Lgl1+/−* mice and found that *Lgl1+/−* mice had increased synapse numbers, impaired social interaction, and increased stereotyped repetitive behavior, suggesting that *Lgl1* is a candidate gene that contributes to the autism-like symptoms of SMS with *Lgl1* deletion. Repetitive behaviors involve the striatum, where *CreER*^*T2*^ is not expressed in the SLICK-H line. This may explain why repetitive behavior defects were only observed in *Lgl1+/−*. There was a slowed habituation to the open field in *Lgl1*+/− and defects in novel object recognition. In summary, loss of both copies of *Lgl1* locally or only one copy of *Lgl1* globally could cause behavioral deficits related to a subset of autism-like symptoms of SMS. Interestingly, *Lgl1* cKO and *Lgl1+/−* animals did not show an increase in grooming behavior during open-field observation or signs of excessive self-grooming while in their home cage that characterizes other ASD-like mouse models (Peça et al., 2011). Lgl1 appears to be important to maintaining proper synapse numbers and normal function of synapses even in adulthood as deleting *Lgl1* at 6 weeks still lead to increase of synapse numbers and changes of synapse structure and function. *Lgl1* may be a key molecule required for synaptic plasticity in adulthood as cKO led to impairment of long-term potentiation. Therefore the loss of *Lgl1* in SMS may underlie the neurobiological basis of behavioral symptoms. We propose that *Lgl1* is a candidate gene contributing to SMS. Our studies also give rise to a mouse model (*Lgl1+/−* mice) for SMS for understanding disease mechanisms and development of treatment. Indeed, we found that blockade of NMDARs rescues social deficits, suggesting that NMDARs may be promising therapeutic targets for SMS with *Lgl1* deletion.

### Limitations of the Study

In Figure 3K, the variability of the data is greater for the control compared to the *Lgl1* cKO. This is a weakness of our data. However, upon closer inspection of the results, all but one of the data points in the control are higher than all data points from the conditional knockout. Therefore, while the data quality is not ideal, we believe that the conclusion would hold even with a larger sample size.

## Methods

All methods can be found in the accompanying Transparent Methods supplemental file.

## Supplemental Information

Document S1. Transparent Methods and Figures S1–S6
