# Supplementary material for: Apical-Basal Polarity Signaling Components, Lgl1 and aPKCs, Control Glutamatergic Synapse Number and Function
Source: iScience. 2019 Sep 9;20:25–41. doi: 10.1016/j.isci.2019.09.005 (PMC6817635; doi:10.1016/j.isci.2019.09.005)
Supplement: Document S1. Transparent Methods and Figures S1–S6 [file mmc1.pdf]

ISCI, Volume *20*

Supplemental Information

Apical-Basal Polarity Signaling

Components, Lgl1 and aPKCs, Control

Glutamatergic Synapse Number and Function

John Scott, Sonal Thakar, Ye Mao, Huaping Qin, Helen Hejran, Su-Yee Lee, Ting Yu, Olga Klezovitch, Hongqiang Cheng, Yongxin Mu, Sourav Ghosh, Valeri Vasioukhin, and Yimin Zou

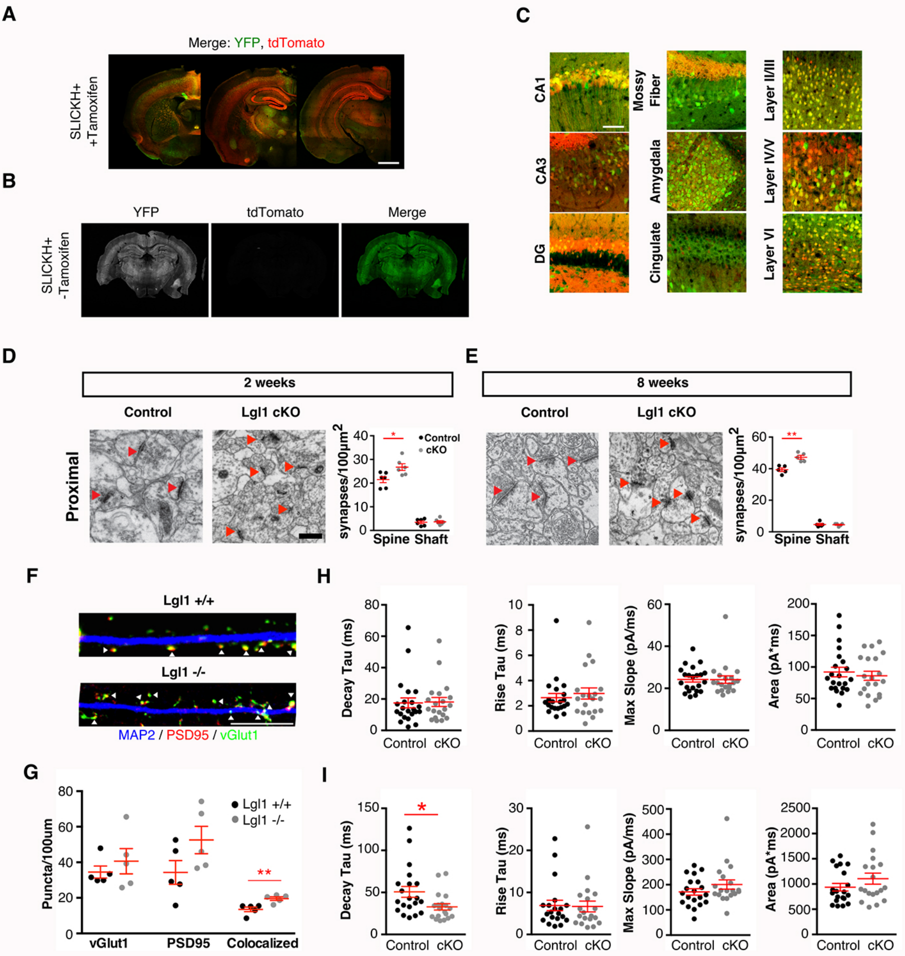

Figure S1 Related to Figure 1: Assessment of conditional deletion and characterization of current kinetics.

(**A**) Confocal images of brain slices from P14 mice injected with Tamoxifen at day P7 and P8 carrying SLICK-H shown by YFP (green) and *ROSA26-tdTomato* (red). Images show regions including the ventral hippocampal commissure and fimbria, dorsal hippocampus, and ventral hippocampus. Scale bar: 1mm. (**B**) Confocal images of brain slices of mice that were not injected with Tamoxifen, but carry SLICK-H and *ROSA26-tdTomato*. Scale bar: 1mm. (**C**) Images of brain regions expressing *Thy-1* cre in SLICK-H positive animals shown by YFP (green) and tdTomato (red). Scale bar: 100 µm. (**D**) EM micrographs taken 50µm from the CA1 pyramidal neuron layer in the Schaffer Collateral Region of P14 mice. Red arrows denote asymmetric synapses. Quantification of asymmetric synapse number: N = 6 control, 6 *Lgl1* cKO animals. (**E**) EM micrographs taken 50µm from the CA1 pyramidal neuron layer in the Schaffer Collateral Region of 8-week old mice. Red arrows denote asymmetric synapses. Quantification of asymmetric synapse number: N = 5 control, 5 *Lgl1* cKO animals. (**F**) Confocal images of dendrites (MAP2; blue) of neurons cultured for 14 DIV showing vGlut1 puncta (green) and PSD95 puncta (red). White arrowheads indicate colocalized pre- and post-synaptic puncta. Scale bar: 10 µm. (**G**) Quantification of puncta within ROIs including secondary dendrites of cultured hippocampal pyramidal neurons. N = 5 control, 5 KO embryos. (**H**) Quantification of kinetics of mEPSCs from slices from P13-15 control and *Lgl1* cKO mice. n = 22 control, 19 *Lgl1* cKO neurons. (**I**) Quantification of kinetics of mIPSCs from slices from P13-15 control and *Lgl1* cKO mice. n = 20 control, 19 *Lgl1* cKO neurons. *p<0.05; **p<0.01.

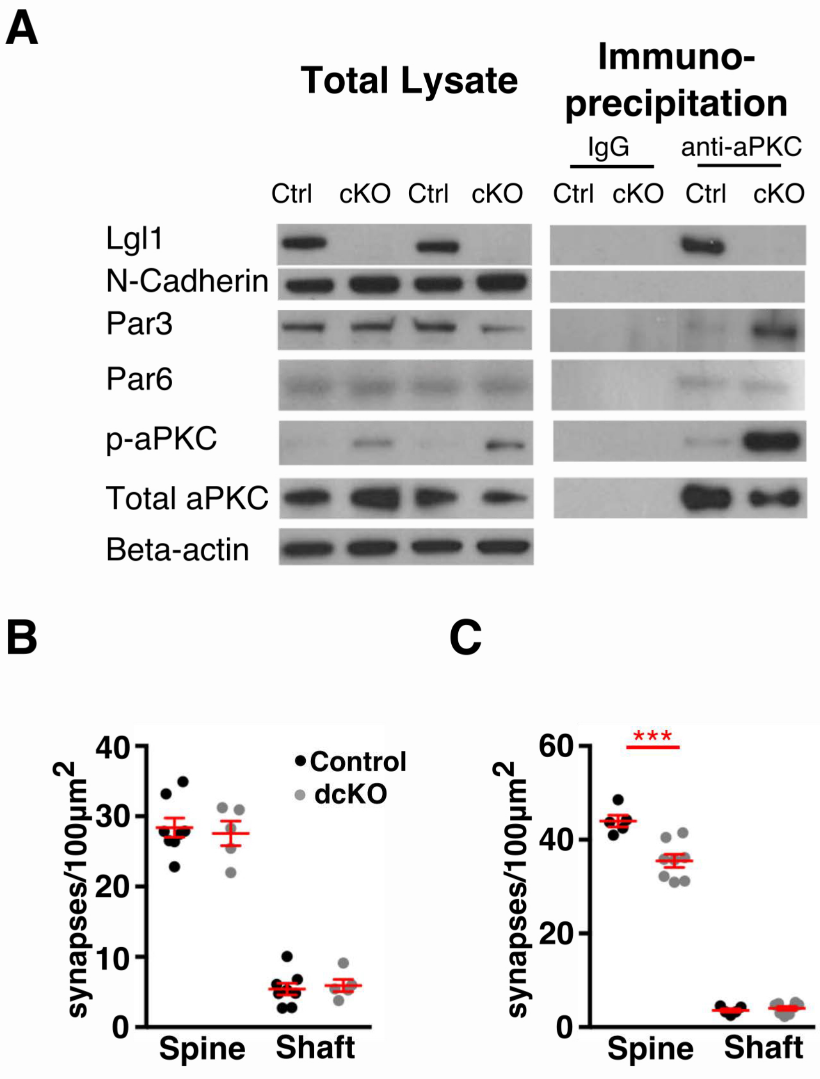

Figure S2 related to Figure 2: *Atypical PKC* overactivation following *Lgl1* conditional deletion and synapse quantification in *aPKC dcKO*. (**A**) Western blots of total cell lysate and immunoprecipitation by anti-aPKC antibody from cultured neuronal progenitors treated with either AdGFP (Ctrl) or AdCre (cKO). (**B**) Quantification of asymmetric synapse number in the region of the Schaffer Collateral 50µm from the CA1 neuron layer in P14 control and *aPKC dcKO* animals. (**C**) Quantification of asymmetric synapse number in the region of the Schaffer Collateral 50µm from the CA1 neuron layer in 8-week old control and *aPKC dcKO* animals. ***p<0.001.

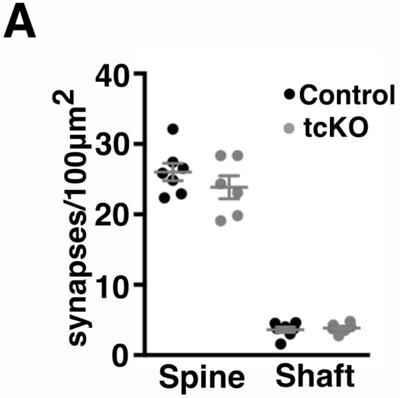

Figure S3 related to Figure 2: Synapse quantification in *Lgl1;PKCι/λ;PKCζ tcKO*. (**A**) Quantification of asymmetric synapse number in the region of the Schaffer Collateral 50µm from the CA1 neuron layer in P14 control and *Lgl1;PKCι/λ;PKCζ tcKO* animals. (**B**) Quantification of asymmetric synapse number in the region of the Schaffer Collateral 50µm from the CA1 neuron layer in 8-week old control and *Lgl1;PKCι/λ;PKCζ tcKO* animals.

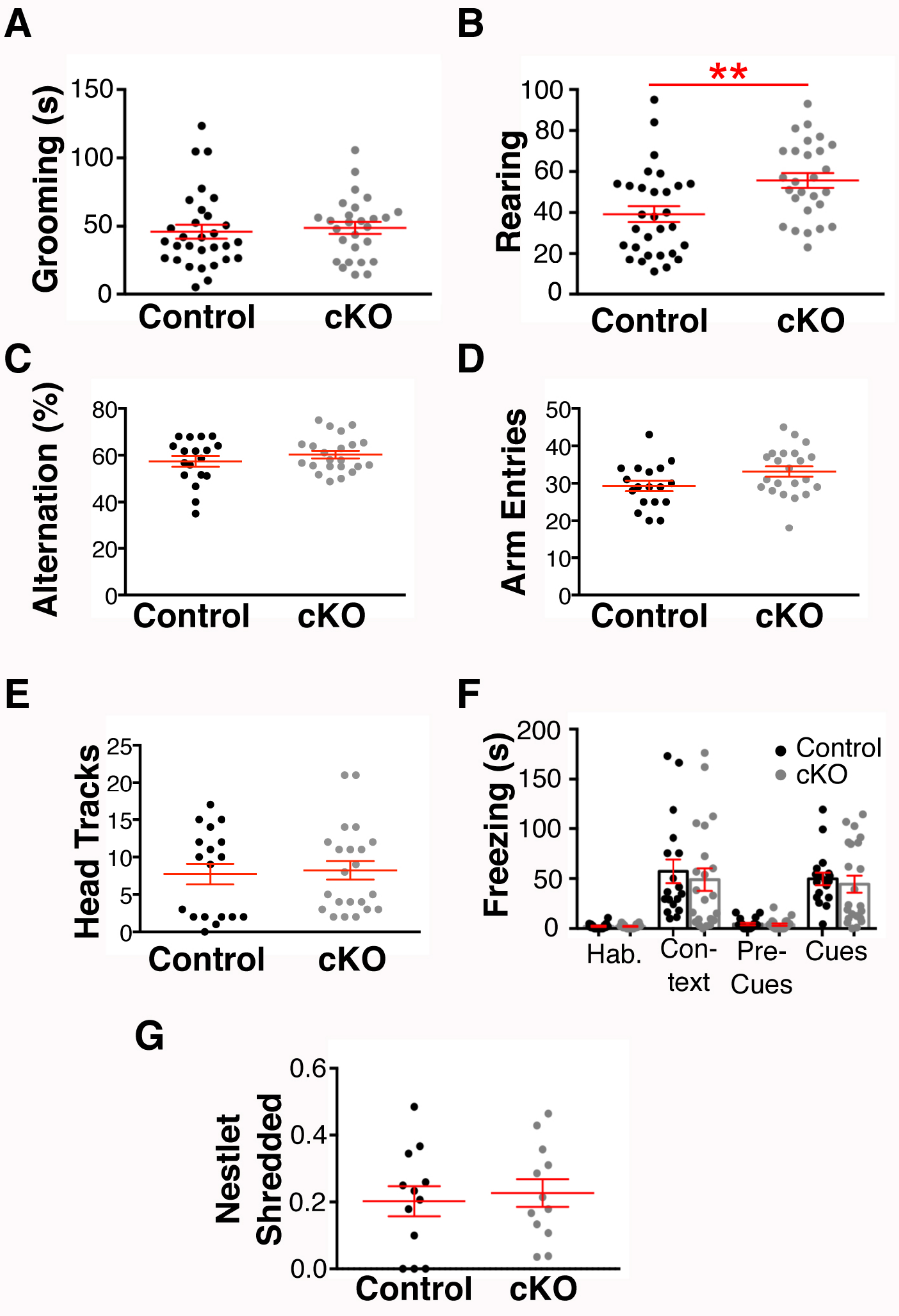

Figure S4 related to Figure 4: Additional behavioral characterization of *Lgl1* deletion at day P7. (**A**) Quantification of time spent self-grooming during the open field test. (**B**) Quantification of rearing counts during the open field test. (**C**) Quantification of spontaneous alternation in the Y-maze test following SLICK-H mediated *Lgl1* deletion at P7/P8. N = 18 control, 22 *Lgl1* cKO. (**D**) Total arm entries in the Y-maze test (not significant; p=0.0560) N = 18 control, 22 *Lgl1* cKO. (**E**) Number of 15-degree head movements during the optomotor response task. N = 18 control, 22 *Lgl1* cKO. (**F**) Quantification of the conditioned fear test following SLICK-H-mediated deletion of Lgl1. N = 18 control, 22 *Lgl1* cKO. (**G**) Quantification of nestlet-shredding activity following conditional *Lgl1* deletion at day P7. N = 12 control, 12 *Lgl1* cKO animals. **p<0.01.

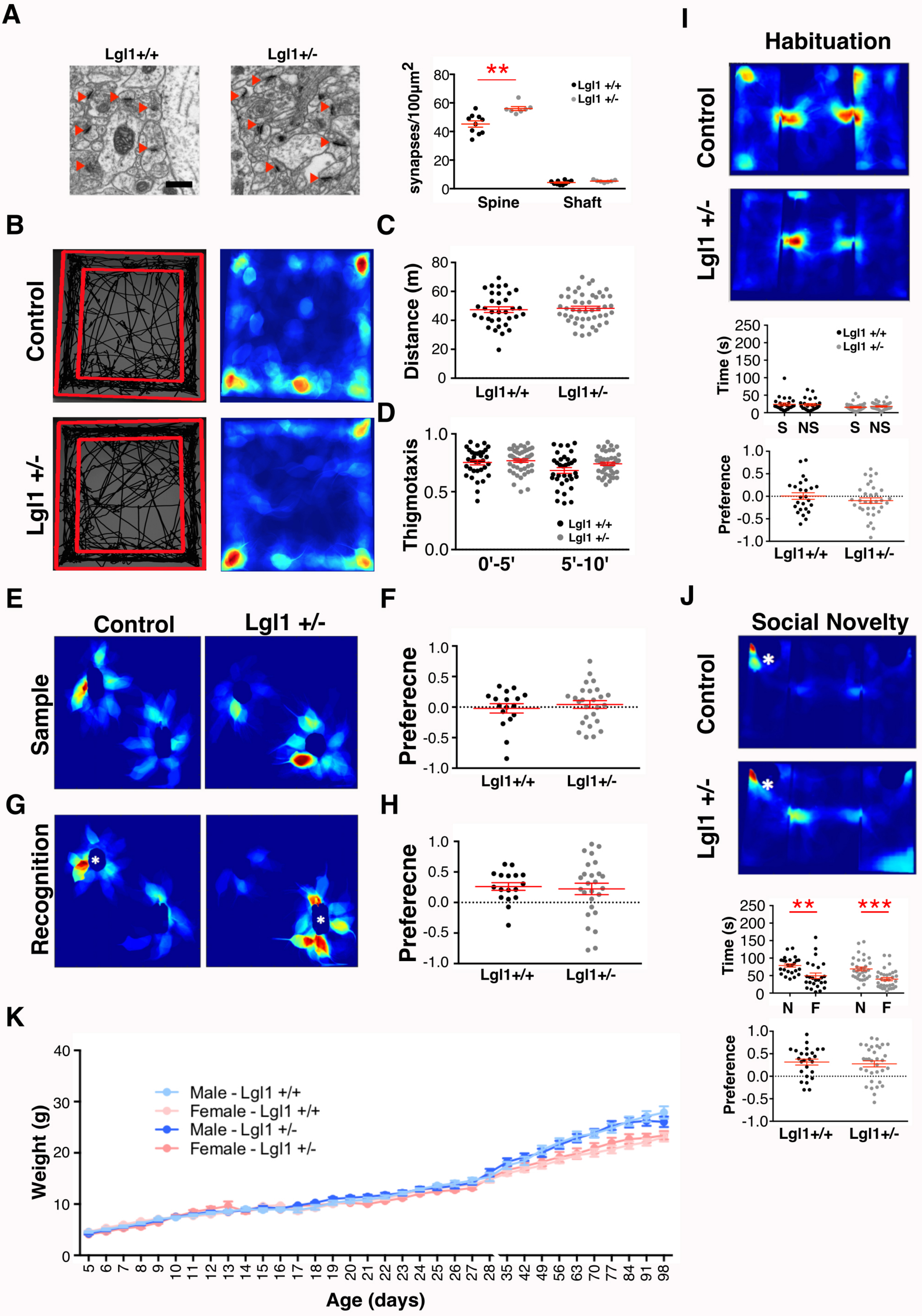

Figure S5 related to Figure 6: Partial preservation of behavioral phenotypes in *Lgl1*$^{+/-}$ mice. (**A**) Quantification of asymmetric synapse number in the region of the Schaffer Collateral 50µm from the CA1 neuron layer in P14 *Lgl1*$^{+/+}$ and *Lgl1*$^{+/-}$ animals. (**B**) Heat maps and Trajectories from *Lgl1*$^{+/+}$ and *Lgl1*$^{+/-}$ in the 10-minute open field (OF) test. (**C**) Quantification of distance travelled during the OF test. N = 34 control, 47 *Lgl1*$^{+/-}$ animals. (**D**) Quantification of Thigmotaxis for control (black) and Lgl1$^{+/-}$ (gray) mice. (**E**) Heat maps of interaction bouts from Control and *Lgl1* cKO from the sample phase of the novel object recognition (NOR) test. (**F**) Quantification of preference for objects during the sample phase. N = 17 control, 26 *Lgl1*$^{+/-}$ animals. (**G**) Heat maps of interaction bouts from the object recognition phase of the NOR test performed 2 minutes after the sample phase. White asterisk indicates location of the novel object. (**H**) Quantification of preference for objects. Positive value indicates preference for novel object. N = 17 control, 26 *Lgl1*$^{+/-}$ animals. (**I**) Heat maps and quantification from the habituation phase of the Social Interaction test. N = 25 control, 33 *Lgl1*$^{+/-}$ animals. (**J**) Heat maps and quantification from the social novelty phase of the SI test. N = 25 control, 33 *Lgl1*$^{+/-}$ animals. (**K**) Comparison of weight gain of *Lgl1*$^{+/+}$ and *Lgl1*$^{+/-}$ mice during development and early adulthood. Note discontinuous X-axis after P28 (N=9-15 male *Lgl1*$^{+/+}$, 9-15 male *Lgl1*$^{+/-}$, 8-15 female *Lgl1*$^{+/+}$, 9-14 female *Lgl1*$^{+/-}$).

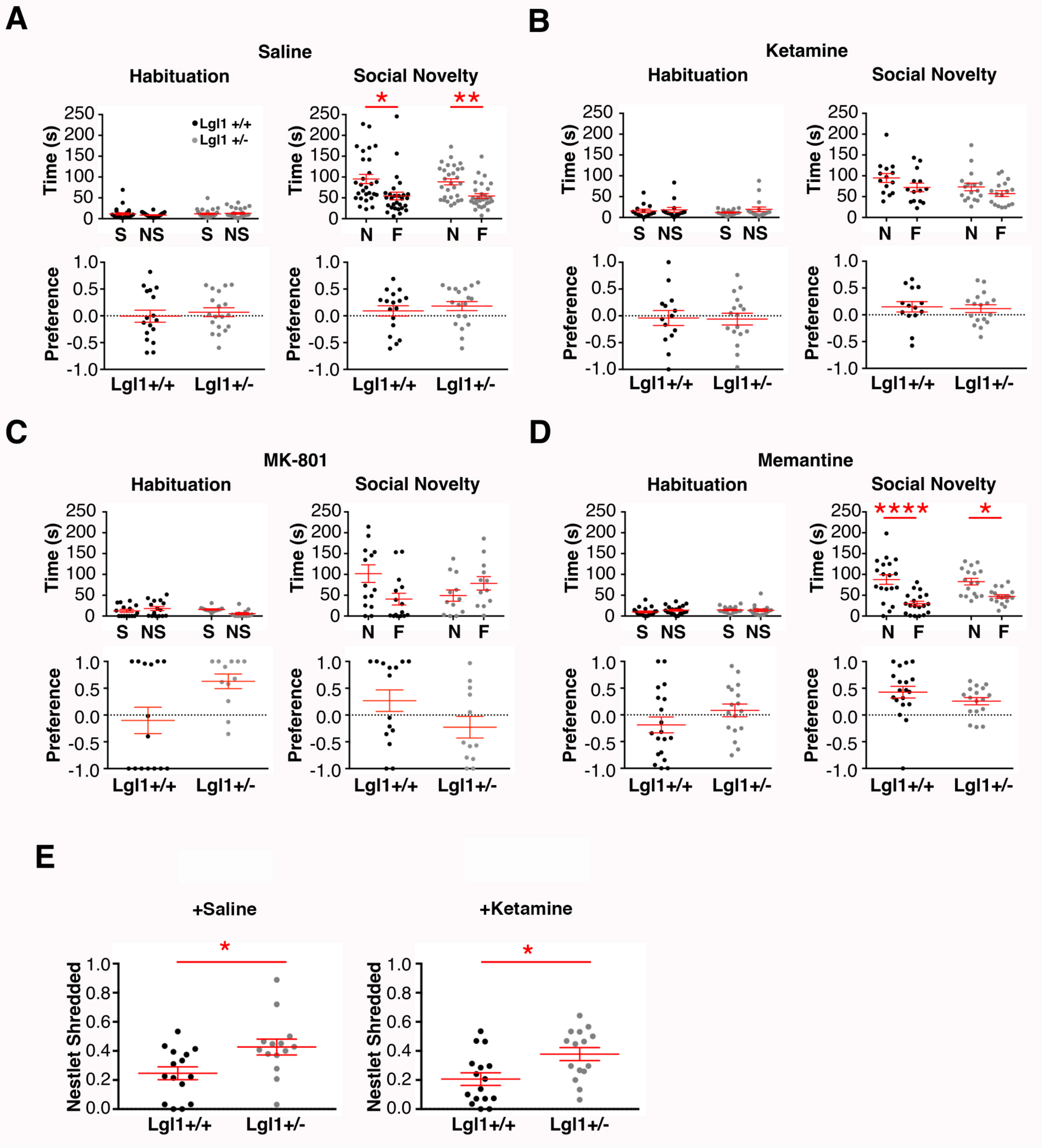

Figure S6 related to Figure 7: Additional phases of social interaction test in saline, ketamine, and MK-801 treated animals. (**A-D**) Quantification of interaction time and preference from the habituation and social novelty phases of the Social Interaction test following administration of the indicated solution. White asterisk indicates location of the novel mouse in the social novelty phase. N = (Saline) 27 *Lgl1*$^{+/+}$, 30 *Lgl1*$^{+/-}$ animals; (ketamine) 14 *Lgl1*$^{+/+}$, 17 *Lgl1*$^{+/-}$ animals; (MK-801) 13 *Lgl1*$^{+/+}$, 14 *Lgl1*$^{+/-}$ animals; (Memantine) 19 *Lgl1*$^{+/+}$, 17 *Lgl1*$^{+/-}$ animals. (**E**) Quantification of nestlet shredding activity following saline or ketamine injection. N = (Saline) 15 *Lgl1*$^{+/+}$, 14 *Lgl1*$^{+/-}$ animals; (ketamine) 15 *Lgl1*$^{+/+}$, 12 *Lgl1*$^{+/-}$ animals *p<0.05; **p<0.01; ****p<0.0001.

Methods

All animal procedures were performed in accordance with protocols approved by the Institutional Animal Care and Use Committee at Univesity of California, San Diego. Both male and female animals were analyzed in roughly equal numbers.

Electron Microscopy

Lgl cKO or *Lgl1*$^{+/-}$ and littermate control mice were anesthetized i. p. with a ketamine/xylazine cocktail, and perfused with modified Karnovsky's fixative (2.5% glutaraldehyde and 2% PFA in 0.15 M sodium cacodylate buffer, pH 7.4) at room temperature. After postfixation in the same solution overnight at 4 °C, whole brains were cut coronally (200 μm) with a vibratome. Sections were postfixed with 1% osmium tetroxide for 1 h on ice. Following en-block staining in 2% uranyl acetate in distilled water for 1-2 h, sections were dehydrated in a graded ethanol series and then treated twice in acetone for 10 min each. Sections were infiltrated in Durcupan resin (Sigma-Aldrich) and were embedded at 60 °C for 48 h. Ultrathin sections from CA1 region were cut and stained with uranyl acetate and Sato's lead. Image acquisition used a FEI Tecnai Spirit G2 Spirit BioTWIN transmission electron microscope equipped with an Eagle 4k HS digital camera (FEI, Hilsboro, OR). 10-15 fields from the distal stratum radiatum (150-200 μm from CA1 pyramidal cell bodies) and proximal stratum radiatum (40-50 μm from CA1 pyramidal cell bodies) were selected under 6800× magnification, and synapses were counted in each field. Synapses that displayed polyribosomes and/or actin bundles were determined to be on the dendritic shaft while synapses lacking those structures were determined to be on the dendritic spines. For ultrastructure analysis, asymmetric synapses from the distal stratum radiatum area were imaged at 18500× magnification. Only asymmetric synapses with a clearly recognized PSD and synaptic cleft were selected. The bouton area, cleft distance, reserve pool vesicles, docked vesicles, PSD width and PSD length were analyzed using Image J. Analysis of ultrastructure was done by experimenters blinded to animal genotype.

Hippocampal primary cell culture

Hippocampal neuron culture was performed as described below (Meffert et al., 2003). Glass coverslips were washed overnight in nitric acid and rinsed thoroughly and washed with 100% ethanol before being placed in a drying oven. The day before cell culturing, coverslips were coated with 80mg/mL Poly-D-Lysine (PDL) overnight at room temperature. Coverslips were washed and coated with 40mg/mL PDL with 2.5mg/mL Laminin for 3 hours at 37°C.
Brains from E18.5 pups were removed in L15 media and hippocampi were dissected and placed in individual tubes. Hippocampi were washed twice with Mg/Ca free sterile PBS (CellGro) and incubated with Trypsin/EDTA solution (0.25% Trypsin, 2.2mM EDTA) for 15 minutes at 37°C. Trypsin was neutralized by the addition of 10% vol/vol heat-inactivated horse serum (Life Technologies). Resulting solutions were pelleted and resuspended in Neurobasal medium supplemented with 2% B27 (Invitrogen), 5% fetal bovine serum (Invitrogen), penicillin/streptomycin (Cellgro), and Glutamax (Invitrogen) and washed twice. Cells density was determined and cells were plated at a density of $2.5x10^4$ cells/cm^2. Half of the growth

medium was exchanged every 3 days. Arabinofuranosyl Cytidine (Ara-C, 4 μM) was added at day 6 *in vitro* to prevent glial cell proliferation. Cultures were grown for 14 days at 37°C in a 5% carbon dioxide atmosphere.

Behavioral assays

For all behavioral assays, testing was performed during the light phase in a dimly lit room (<100 lux) with indirect lighting on the testing area unless otherwise noted. Both male and female animals were tested in behavior tests.

Open field test:
The open field test is performed in a plastic 40cm x 40cm acrylic box (Stoetling) with dark walls with a non-reflective base plate. Spatial cues are placed on one wall of the field. Mice are placed in the center of the field and allowed to explore freely for 10 minutes. Mice are scored for distance travelled, time in the inner and outer regions of the field, time spent self-grooming, rearing activity, and jumping activity. Outer region is defined as the region within 5 centimeters of the edge of the field. A 60-minute variant of the test was applied to the *Lgl1*$^{+/-}$ mice and littermate controls with all aspects of the testing area identical to the 10-minute test. Animals that performed the 10-minute test were not used for the 60-minute test in order to avoid effects of prior exposure to the testing apparatus.

Novel object recognition test:
The novel object recognition test was performed as described below (Antunes and Biala, 2012) immediately following a 10-minute exploration period in an empty apparatus. Animals are placed in a field containing two copies of a novel object and allowed to explore freely. After the sample phase, animals were removed from the field and the objects were replaced with one copy of the previously explored object and a novel object in the same positions in the field as during the sample phase. Animals were allowed to explore freely for 5 minutes. For scoring of interaction time, ROIs containing the target objects were selected in MATLAB. ROIs were selected by experimenters blinded to the animal genotypes or virus treatment.

Social interaction test:
The social interaction test was performed as previously described below (Yang et al., 2011). The sociability apparatus (Stoetling) has 3 20cm x 40 cm chambers and 2 target enclosures 7cm in diameter and 15cm tall. During testing, the apparatus was surrounded by opaque panels to prevent mice from seeing out of the apparatus.

Animals were placed in the center chamber of the 3-chambered sociability apparatus with the doors between chambers closed for 10 minutes to habituate to the testing conditions. The doors were removed and mice were allowed to explore the full field for 10 minutes. Non-littermate strain- and age-matched target mice from a separate cage were introduced during the social interaction phase of the test. For the social novelty phase, target mice from the social interaction phase were kept in the same location and a novel target mouse was introduced to the opposite chamber. For scoring of interaction time, ROIs containing the target mouse and

novel object were selected in MATLAB. ROIs were selected by experimenters blinded to the animal genotypes.

Scoring for distance travelled and thigmotaxis during the open field test, as well as time spent interacting with objects in the Novel Object Recognition test and time spent interacting with target mice and objects in the Social Interaction test was performed using Autotyping 15.04 in MATLAB 2014b (Patel et al., 2014)(MathWorks).

Nestlet shredding:
Mice were removed from their home cage and placed individually into a clean novel cage containing a weighed piece of cotton nesting material of approximately 2.8g. Mice were left in the cage for 60 minutes. At the end of the testing period, mice were returned to their home cage. Shredded material was separated from the undisturbed portion, which was weighed(Li et al., 2006).

Electrophysiology

For *Lgl1* cKOs and littermate controls at P13–15, mice were anesthetized by isofluorene. Mice were decapitated, and their brains were quickly removed and placed in ice-cold dissection buffer containing the following (in mM): 87 NaCl, 2.5 KCl, 1.25 NaH_2PO_4, 25 $NaHCO_3$, 20 glucose, 75 sucrose, 0.5 $CaCl_2$, and 7 $MgCl_2$, pH 7.35. Transverse hippocampal slices were cut in 300 μm-thick sections on a vibratome (Lieca VT1200). Slices were allowed to recover at 35°C for 20 min and then at room temperature for 1 to 6 hr in carbogenated ACSF, containing (in mM) 124 NaCl, 2.5 KCl, 24 $NaHCO_3$, 1.2 NaH_2PO_4, 2 $CaCl_2$, 2 $MgCl_2$ and 12.5 glucose, 5 HEPES pH 7.4. Individual slices were transferred to a recording chamber and then continuously perfused at a rate of 2–3 ml/min with ACSF containing 1 μM TTX (Tocris) and either 20 μM gabazine or 20 μM CNQX for mEPSC or mIPSC recordings, respectively. CA1 pyramidal cells were visualized by infrared differential interference microscopy.

For mEPSCs, whole-cell recordings were made using 3–5 MΩ pipettes filled with an internal solution that contained (in mM): 145 CH3O3SCs, 5 NaCl, 10 HEPES, 5 EGTA, 0.3 Na_2GTP and 4 MgATP, (pH was adjusted to 7.3 and osmolarity was maintained at 280–290 mOsm). For mIPSC recordings, whole-cell recordings were made using 3–5 MΩ pipettes filled with an internal solution that contained (in mM): 135 CsCl, 4 $MgCl_2$, 0.1 EGTA, 10 HEPES, 2 MgATP, 0.3 NaGTP, 10 Na_2Phosphocreatine (pH was adjusted to 7.3, and osmolarity was maintained at 280–290 mOsm).

Cells were voltage clamped at -70 mV. Recordings started after 5 minutes to allow for stabilization of the established whole-cell configuration. Signals were recorded with a 5× gain, low-pass filtered at 2 kHz and digitized at 5 kHz (Molecular Devices Axopatch 200B) with pCLAMP 10 software (Molecular Devices); analysis was performed with Clampfit (pCLAMP). The automatic detection was verified post hoc by visual inspection. Values are presented as mean ± SEM. Mann-Whitney U-statistic test was used to compare changes with the control.

For LTP induction, transvers hippocampus slices were harvested from 9-10-week-old mice. Slices were recovered for at least 1 hour before moving to recording chamber in aCSF (in mM): NaCl 119, KCl 2.5, NaH2PO4 1, NaHCO3 26.2, Glucose 11, MgCl2 1.3 and CaCl2 2.5 bubbled with 95% O2 and 5% CO2. Stimulus was given at 0.033 Hz to evoke field potential and the resistance of glass electrode was 1-2 MΩ with aCSF as internal solution. Input-Output were recorded by gradually increasing the strength of stimulus. After 30min stable baseline recording, LTP was induced by HFS or TBS. HFS was 100 pulses at 100 Hz. TBS contained four trains of theta bursts with 20 second intervals, each train had 10 burst with 0.2 interval.

For AMPA/NMDA ratio determination, a broken glass pipette was used as a stimulus electrode. The resistance of patch the clamp pipette was 3-5 MΩ. Internal solution: (in mM) CsMeSO4 115, CsCl 20, Na phosphocreatine 10, MgCl2 2.5, Na2ATP 4, Na3GTP 0.3, EGTA 0.6, HEPES 10 and QX-314-Cl 5. A cut was made between CA1 and CA3 to avoid epilepsy events. The position of stimulus electrode was gently adjusted to evoke a single peak EPSC. For AMPA current, holding potential was set at -70mV, and for NMDA current, holding potential changed to +40mV. We quantified NMDA current as the peak 100ms after stimulus artifact, where there is no AMPA current.

Biochemical fractionation

Subcellular fractionation was performed as previously described below (Cohen et al., 1977) with modifications. Forebrains from P14 wild type mice were homogenized to 10% (wt/vol) in ice cold 0.32M sucrose buffer containing 1mM MgCl2, 0.5mM CaCl2, 1mM NaHCO3 and protease inhibitors using 16 strokes with a glass dounce. The homogenates were spun at 710g for 30 min at 4C to pellet out nuclei and large debris. The supernatant was further centrifuged at 13800g for 10 min at 4C to get pellets (P2). P2 pellets were resuspended in the sucrose buffer and layered on top of a discontinuous sucrose gradient containing 1.0M and 1.4M sucrose in 4mM HEPES buffer pH 7.4. The gradient was centrifuged at 82500g for 1h at 4C. Synaptosomes were recovered from the cloudy band between 1.0M and 1.4M sucrose, resuspended in 1mM $NaHCO_3$ (1:9 vol/vol), and lysed by hypo osmotic shock using 3 strokes with a glass dounce. The lysates were then incubated with an equal volume of 0.32M sucrose buffer containing 1% Triton-X shaking at 4C for 15 min and spun at 82500g for 1h. The PSD fraction was obtained from the resulting pellet. The synaptic membrane fraction (SMF) was precipitated from the supernatant using the methanol/chloroform/H2O method(Wessel and Flügge, 1984). Both the PSD fraction and SMF were solubilized in 3% SDS for western blot analysis.

Western Blot

Protein samples were separated by polyacrylamide gel electrophoresis on 5% and 8% acrylamide gels and wet transferred to Immobilon membranes (Millipore). TBST with 5% non-fat dry milk (Apex) was used to block and primary antibody was diluted in blocking buffer for overnight incubation at 4°C. Blots were washed with TBST and incubated with HRP-conjugated secondary antibody for 2 hours at room temperature. Bands were visualized using West Pico

Chemiluminescent Substrate and exposed to film at intervals ranging from 30 seconds to 2 hours.

Antibodies

Primary antibodies were mouse anti-Lgl1 (gift of Valeri Vasioukhin), mouse anti-Lgl1 (Novus), Goat anti Vangl2 Antibody (N-13, Sant cruz, sc-46561), goat anti-JAM-C (R&D Systems), mouse anti-GAPDH (Abcam), and rabbit anti-GFP (Invitrogen), and chicken anti-MAP2 (Abcam). Secondary antibodies used in western blot were HRP-conjugated donkey anti-mouse, anti-goat, and anti-rabbit. For immunofluorescence, AF568-conjugated Donkey anti-Mouse (Invitrogen), AF488-conjugated Donkey anti-Chicken (Jackson Immunoresearch), AF488-conjugated Donkey anti-Rabbit (Invitrogen), AF647-conjugated Donkey anti-Rabbit (Invitrogen).

Cell filling and spine analysis

Mice were perfused, sectioned, and labeled with AlexaFluor Hydrazide as described below (Dumitriu et al., 2011). SLICK-H:*Lgl1*$^{flox/flox}$ pups were i. p. injected with tamoxifen at P7-8. At P14, pups were anesthetized with a ketamine/xylazine cocktail followed by transcardial perfusion with room temperature 4% PFA, postfixed for one hour in 4% PFA, vibratome sectioned at 100 μm, and then postfixed again for 10 minutes. AlexaFluor Hydrazide 555 (Invitrogen, 10 mM in 200 mM KCl) was injected into dendritic segments ionophoretically by filling the cell with fluorescent dye. Sections were briefly fixed for 15 minutes to preserve the fluorescent label. Immunolabeling with GFP antibody (Invitrogen) was used to confirm that the fluorescently filled dendrite is YFP+. 30-40 μm segments of CA1 oblique apical dendrites, which are located 100-200 μm from the CA1 pyramidal neuron cell bodies in the stratum radiatum, were analyzed. These spines represented the postsynaptic structures of the Schaffer collateral-CA1 synapses.

The maximum spine length and head width were measured manually with Image J (NIH) to characterize spine shape as described below (Bochner et al., 2014; Calfa et al., 2012; Harris et al., 1992; Tang et al., 2014; Vogel-Ciernia et al., 2013). Spines with heads equal to or less than the head width were categorized as “thin”, and those with heads greater than the neck width were categorized as “mushroom”. Spines without a neck that had a width longer than their lengths were termed “stubby”. Spines with one neck and a branch point resulting in 2 heads were termed “branched”. Protrusions not clearly seen or with lengths > 5 μm were excluded from analysis. Both image acquisition and morphometric analyses were done by experimenters blinded to the genotypes of the animals. Results were compared between littermates and then pooled by genotypes to assess the influence of prenatal care.

Tissue preparation

Animals were anesthetized i. p. with a ketamine/xylazine cocktail and perfused with ice-cold PBS followed by 4% Paraformaldehyde (PFA). Brains were post-fixed in 4% PFA overnight and moved to 30% sucrose until equilibrated. Frozen section embedded in OCT medium and Sucrose were sectioned at 40μm on a Leica CM 3050 S cryostat. Slides were stored at 4°C until immunostaining.

Immunostaining

For tissue sections, slides were rehydrated with 2 washed of PBS and permeabilized with PBS + 0.3% Triton X-100. Slides were blocked with 5% normal donkey serum for 2 hours at room temperature. Primary antibody was incubated at 4°C overnight and secondary antibody was incubated for 2 hours at room temperature. Slides were coverslip with Fluoromount-G (Southern Biotech) and stored at 4°C.

For cultured neurons, glass coverslips with primary cell cultures were washed with PBS and permeabilized with PBS + 0.1% Triton X-100. Coverslips were blocked with PBS containing 2% BSA, 2% Fetal Bovine Serum, and 0.1% Triton X-100. Primary antibody was incubated at 4°C overnight and secondary antibody was incubated for 2 hours at room temperature. Coverslips were inverted onto slides with Fluoromount-G (Southern Biotech) and stored at 4°C.

Statistical Analysis

Statistical analysis was performed using Prism 6 (GraphPad). Student's T-test with Welch's correction was used for comparison between control and Lgl1 mutant animals unless otherwise noted. One-way ANOVA with Tukey's multiple comparison test was used to test for statistical differences in interaction time during the social interaction test. Graphs of quantitative data present individual data points for animals with mean and standard error of the mean indicated unless otherwise noted. The Kolmogorov-Smirnov test was used to compare distribution of measurements of synaptic ultrastructure.

References:

Antunes, M., and Biala, G. (2012). The novel object recognition memory: neurobiology, test procedure, and its modifications. Cogn Process *13*, 93-110.

Bochner, D., Sapp, R., Adelson, J., Zhang, S., Lee, H., Djurisic, M., Syken, J., Dan, Y., and Shatz, C. (2014). Blocking PirB up-regulates spines and functional synapses to unlock visual cortical plasticity and facilitate recovery from amblyopia. Sci Transl Med *6*, 258ra140.

Calfa, G., Chapleau, C.A., Campbell, S., Inoue, T., Morse, S.J., Lubin, F.D., and Pozzo-Miller, L. (2012). HDAC activity is required for BDNF to increase quantal neurotransmitter release and dendritic spine density in CA1 pyramidal neurons. Hippocampus *22*, 1493-1500.

Cohen, R., Blomberg, F., Berzins, K., and Siekevitz, P. (1977). The structure of postsynaptic densities isolated from dog cerebral cortex: I. overall morphology and protein composition. Journal of Cell Biology *74*, 181-203.

Dumitriu, D., Rodriguez, A., and Morrison, J. (2011). High-throughput, detailed, cell-specific neuroanatomy of dendritic spines using microinjection and confocal microscopy. Nature Protocols *6*, 1391-1411.

Harris, K.M., Jensen, F.E., and Tsao, B. (1992). Three-dimensional structure of dendritic spines and synapses in rat hippocampus (CA1) at postnatal day 15 and adult ages: implications for the maturation of synaptic physiology and long-term potentiation. Journal of Neuroscience *12*, 2685-2705.

Li, X., Morrow, D., and Witkin, J. (2006). Decreases in nestlet shredding of mice by serotonin uptake inhibitors: Comparison with marble burying. Life Sci *78*, 1933-1939.

Meffert, M., Chang, J., Wiltgen, B., Fanselow, M., and Baltimore, D. (2003). NF-kappa B functions in synaptic signaling and behavior. Nature Neuroscience *6*.

Patel, T.P., Gullotti, D.M., Hernandez, P., O'Brien, W.T., Capehart, B.P., Morrison, B., Bass, C., Eberwine, J.E., Abel, T., and Meaney, D.F. (2014). An open-source toolbox for automated phenotyping of mice in behavioral tasks. Frontiers in Behavioral Neuroscience *8*, 1-16.

Tang, G., Gudsnuk, K., Kuo, S., Cotrina, M., Rosoklija, G., Sosunov, A., Sonders, M., Kanter, E., Castagna, C., Yamamoto, A.*, et al.* (2014). Loss of mTOR-dependent macroautophagy causes autistic-like synaptic pruning deficits. Neuron *83*, 1131-1143.

Vogel-Ciernia, A., Matheos, D., Barrett, R., Kramár, E., Azzawi, S., Chen, Y., Magnan, C., Zeller, M., Sylvain, A., Haettig, J.*, et al.* (2013). The neuron-specific chromatin regulatory subunit BAF53b is necessary for synaptic plasticity and memory. Nat Neurosci *16*, 552-561.

Wessel, D., and Flügge, U. (1984). A method for the quantitative recovery of protein in dilute solution in the presence of detergents and lipids. Anal Biochem *138*, 141-143.

Yang, M., Silverman, J., and Crawley, J. (2011). Automated Three-Chambered Social Approach Task for Mice. Curr Protoc Neurosci *8*, Unit 8.26.
